# The Nairovirus Nairobi Sheep Disease Virus/Ganjam Virus Induces the Translocation of Protein Disulphide Isomerase-Like Oxidoreductases from the Endoplasmic Reticulum to the Cell Surface and the Extracellular Space

**DOI:** 10.1371/journal.pone.0094656

**Published:** 2014-04-08

**Authors:** Lidia Lasecka, Michael D. Baron

**Affiliations:** The Pirbright Institute, Pirbright, Woking, Surrey, United Kingdom; University of Liverpool, United Kingdom

## Abstract

Nairobi sheep disease virus (NSDV) of the genus *Nairovirus* causes a haemorrhagic gastroenteritis in sheep and goats with mortality up to 90%; the virus is found in East and Central Africa, and in India, where the virus is called Ganjam virus. NSDV is closely related to the human pathogen Crimean-Congo haemorrhagic fever virus, which also causes a haemorrhagic disease. As with other nairoviruses, replication of NSDV takes place in the cytoplasm and the new virus particles bud into the Golgi apparatus; however, the effect of viral replication on cellular compartments has not been studied extensively. We have found that the overall structure of the endoplasmic reticulum (ER), the ER-Golgi intermediate compartment and the Golgi were unaffected by infection with NSDV. However, we observed that NSDV infection led to the loss of protein disulphide isomerase (PDI), an oxidoreductase present in the lumen of the endoplasmic reticulum (ER) and which assists during protein folding, from the ER. Further investigation showed that NSDV-infected cells have high levels of PDI at their surface, and PDI is also secreted into the culture medium of infected cells. Another chaperone from the PDI family, ERp57, was found to be similarly affected. Analysis of infected cells and expression of individual viral glycoproteins indicated that the NSDV PreGn glycoprotein is involved in redistribution of these soluble ER oxidoreductases. It has been suggested that extracellular PDI can activate integrins and tissue factor, which are involved respectively in pro-inflammatory responses and disseminated intravascular coagulation, both of which manifest in many viral haemorrhagic fevers. The discovery of enhanced PDI secretion from NSDV-infected cells may be an important finding for understanding the mechanisms underlying the pathogenicity of haemorrhagic nairoviruses.

## Introduction

Nairobi sheep disease virus (NSDV) belongs to genus *Nairovirus* of the family *Bunyaviridae* and causes a severe disease characterised by fever and haemorrhagic gastroenteritis in sheep and goats with a mortality rate up to 90% in a susceptible population [1], [2]. Nairobi sheep disease was first reported in 1910 in Nairobi, Kenya and, in 1917, NSDV was shown to be the causative agent of the disease by Montgomery and colleagues [2]. The virus is endemic in East and Central Africa [2]–[7] and an Asian virus causing the same disease in India is called Ganjam virus (GV) (reviewed in [8]). Based on genetic and serological studies, these viruses were identified as different isolates of the same virus [9], [10].

NSDV does not appear to be contagious and the virus needs to be transmitted by ticks in natural infection [2]. The virus is primarily transmitted by hard (*Ixodid*) ticks, with *Rhipicephalus appendiculatus* being the main vector in Africa [4] and *Haemaphysalis intermedia* the main vector in India [11]–[13]. Sheep and goats are the only known mammalian reservoir for NSDV [3], [4]; other livestock (e.g. cattle, horses) are refractory to the disease [2]. While the virus has a limited effect on animals bred in the enzootic areas due to the development of immunity by these animals while they are still protected by maternal antibodies, NSDV causes large economic losses during transport of animals through enzootic areas or during introduction of new livestock to these areas [2], [14]–[16]. Currently there is no safe vaccine [17].

NSDV is closely related to a human pathogen, Crimean-Congo haemorrhagic fever virus (CCHFV), which causes viral haemorrhagic fever with an average mortality rate of 30% (reviewed in [18], [19]). After Dengue virus (DENV), CCHFV is the second most widespread of the arboviruses pathogenic to humans [20]–[28]. The disease caused by CCHFV in humans is similar to that caused in sheep and goats by NSDV infection [2], [29], and is characterised by fever, myalgia, superficial and internal haemorrhage, abdominal pain and diarrhoea [30]–[33]. While work on CCHFV is limited to biosafety level (BSL) 4 laboratories and restricted by lack of a natural animal model to study the disease, NSDV may act as a suitable model to study haemorrhagic nairoviruses.

Nairoviruses are enveloped viruses which appear spherical in the electron microscope, with a diameter of approximately 100 nm [34]–[36]; the viral genome consists of three negative-sense RNA segments [37]–[39] which are encapsidated by the viral nucleoprotein forming, together with the viral RNA, the ribonucleoprotein (RNP) [37], [40]–[45]. The three RNA segments are called small (S), medium (M) and large (L) and encode respectively the nucleoprotein (N) [37], [42], [46], the viral structural (Gn and Gc) and non-structural glycoproteins [37], [47]–[50], and the RNA-dependent RNA polymerase (i.e. the L protein), which is associated with each RNP [17], [51]–[53].

Replication of nairoviruses occurs in the cytoplasm, where the N and L proteins are also synthesised [54], [55], while the viral glycoproteins are generated on endoplasmic reticulum (ER)-associated ribosomes [49], [50], [56]–[59]. Newly synthesised virions bud into the Golgi and are transported, probably through the secretory pathway, to the plasma membrane, where the virions exit the infected cell after fusion of the carrier vesicle with the plasma membrane [35], [36], [60]. The M segment mRNA is thought to be translated as a single polyprotein, which is further processed by the cellular signal protease [48], [50] to generate the glycoproteins PreGn and PreGc; in CCHFV these precursors are further processed into mature glycoproteins Gn and Gc in a process which employs other cellular proteases [50], [57]–[59]. Since the glycoproteins of nairoviruses mature in the ER and Golgi, and newly generated virions bud in the Golgi, nairoviruses would be expected to affect the secretory pathway. However the effect of nairovirus replication on specific cellular compartments has not been studied; in this study we used specific antibodies in combination with laser scanning confocal microscopy to study the effects of NSDV on the secretory pathway of infected cells. We observed that while NSDV replication has no obvious effect on the structure of the ER, the ER-Golgi intermediate compartment (ERGIC) or the Golgi, it induces redistribution of luminal ER oxidoreductases protein disulphide isomerase (PDI) and ERp57, proteins which are involved in the formation and rearrangement of disulphide bonds during protein folding. In NSDV-infected cells, both PDI and ERp57 are translocated from the ER to the cell surface and to the medium surrounding the infected cells, a process which seems to depend on the NSDV glycoprotein precursor PreGn.

## Materials and Methods

### Cells and viruses

All cell culture was carried out as previously described [29], with the exception of CJE102 cells. These cells are a caprine endothelial cell line derived in 1999 from circulating endothelial cells isolated from a naïve Creole goat that was part of a herd kept for research purposes by the French Centre for Agricultural Research for Development (CIRAD). The blood was taken for the purpose of isolating the cells. All animal sampling was conducted according to internationally approved OIE standards, under personal and institutional authorizations set forth by the director of the veterinary services of Guadeloupe on behalf of the Prefect of Guadeloupe (authorization number: A-971-18-01) and according to the regulations in the Guide to the Care and Use of Experimental Animals provided by the French Ministry of Agriculture, which follow the relevant EU directive [61]. Cell line CJE102 was the kind gift of Dr Nathalie Vachiéry, Département EMVT du CIRAD, Domaine de Duclos, Prise d'Eau, 97170 Petit Bourg, Guadeloupe. The cells were cultured in Glasgow Modified Eagle's Medium (GMEM) containing 10% foetal calf serum (FCS), penicillin (100 U/ml), streptomycin sulphate (100 mg/ml), 2 mM L-glutamine and 5% tryptose phosphate broth.

The highly tissue culture passaged NSDV isolate from Uganda (NSDVu) (ND66-PC9), which was obtained from Dr Piet van Rijn, Central Veterinary Institute of Wageningen, Netherlands, at the 75^th^ tissue culture passage, and the pathogenic GV isolate (NSDVi) (IG619, TVPII 236), which was obtained from Professor Robert B. Tesh, The World Reference Center for Emerging Viruses and Arboviruses at the Galveston National Laboratory, University of Texas Medical Branch, Galveston, Texas, USA, were also already described by us [29].

### Plasmids

All DNA manipulation was done using standard methods; plasmids were grown in *Escherichia coli* DH5α and DNA was purified using CsCl gradients. Plasmids pcDNA6-GV-N and pcDNA6-GV-La plasmids have been previously described [61]. Plasmid pCAGGS-MCSII was the gift of Professor Adolfo Garcia-Sastre, Mount Sinai School of Medicine, New York, USA. The pCAGGs_MCSII_V5 construct was generated by insertion of V5 tag sequence into the multiple cloning site (MCS) of pCAGGS-MCSII; pCAGGs_MCSII_V5 was further used to generate pCAGGs_MCSII_PreGn_V5, pCAGGs_MCSII_NS_M__V5 and pCAGGs_MCSII_PreGc_V5. These constructs were generated by amplification of appropriate fragments of the GV (i.e. NSDVi) M ORF via PCR, PreGn (nucleotides 1-2310 of GV M ORF), NS_M_ (nucleotides 2248-2781) or PreGc (nucleotides 2719-4872), using specific primers (PreGn Fwd: TCTCAAGAATTCGACGACATGGCATTAGTGGCAAAGGG; PreGn Rev: TATCATATCGATGCCCTGCACAGGAGATATAGT; NS_M_ Fwd: TTCCTAGAATTCAACATGAGATTGTCTTGGTTCGGCGTG; NS_M_ Rev: ATTTAGAGATCTGGCTGCTGCAGGCTTCAAGATAATTA; PreGc Fwd: TTCCTTGAATTCGCCACCATGGTTGTGAGCACACTTATTTTAACAGT; PreGc Rev: TATCATATCGATGGCAACCTCTTTAACTGCTTTTC) and pcDNA-GV-M [62] as template. These fragments were then inserted upstream of, and in frame with, the V5-tag of pCAGGs_MCSII_V5. All PCRs were performed using proofreading polymerase (KOD; Novagen). All inserts were sequenced completely.

### Antibodies

The generation of specific rabbit antisera to the amino-terminus of the GV (NSDVi) N protein will be described in detail elsewhere. Briefly, bacterially expressed proteins consisting of the first 167 amino acids of the viral N protein or the first 169 amino acids of the viral L protein were used by a commercial company to immunise four rabbits and the resulting sera optimised for immunofluorescence microscopy. Mouse monoclonal recognising the denatured form of the PreGn glycoprotein from NSDVi, but not from NSDVu, was obtained from AbMart. For detection of cellular markers the following antibodies were used: mouse monoclonal anti-PDI [clone 1D3] was obtained from Bioquote, mouse monoclonal anti-PDI [clone RL90] and anti-calnexin [clone AF18] antibodies were purchased from Abcam; mouse monoclonal Anti V5-Tag:Alexa Fluor 488 was obtained from AbDSerotec, mouse monoclonal anti-α-tubulin from Sigma, mouse monoclonal anti-GM130 from BD Bioscience, mouse monoclonal anti-PCNA (PC10) from Santa Cruz Biotechnology. Alexa Fluor 488 goat anti-mouse IgG (H+L), Alexa Fluor 568 goat anti-mouse IgG (H+L), Alexa Fluor 488 goat anti-rat IgG (H+L), Alexa Fluor 568 goat anti-rabbit IgG (H+L) and Zenon Rabbit IgG Labelling Kit were purchased from Life Technologies. Rat anti-p102 antibody (clone 23C), was prepared by the Institute of Cancer Research [63] and was kindly given to us by Dr Philippa Hawes, Pirbright Institute; antibody to ERp57 has been described previously [64] and was kindly given to us by Dr Christopher Netherton, Pirbright Institute.

### Virus infection and transfection of eukaryotic cells

Vero cells or CJE cells were plated at an initial seeding density of 4 x10^4^ cells/well in 12-well plates. On the next day, cells were infected with NSDVi or NSDVu at a multiplicity of infection indicated for each experiment. The virus inoculum was removed after one hour, cells were washed once with phosphate buffered saline (PBS) and fresh medium was added. The infected cells were further incubated for the times indicated for each individual experiment.

All transfections were carried out with TransIT-LT1 (Mirus) according to the manufacturer's instructions. A ratio of 2 μl TransIT-LT1 reagent per 1 μg of DNA was used; Vero cells were plated as described above, usually 1 day prior to transfection.

### Immunofluorescence

Vero cells were plated as described above on 18 mm-diameter coverslips prior to infection or transfection. At the indicated times, cells were fixed with 3% paraformaldehyde (PFA) and then briefly permeabilised with ice-cold 100% methanol. The fixed and permeabilised cells were blocked with 0.2% porcine gelatine for at least 30 min before processing for antibody staining.

For detection of surface proteins, Vero cells were fixed with 3% PFA, blocked for 30 min with 0.2% porcine gelatine and incubated with primary antibody against PDI or ERp57. After washing with PBS to remove unbound antibodies, the cells were again fixed with 3% PFA, treated briefly with ice-cold 100% methanol and again blocked with the 0.2% porcine gelatine. Such permeabilised cells were incubated with anti-NSDV antibodies, and then proteins were visualised with fluorophore-conjugated secondary antibodies.

Initial confocal images were obtained using sequential scanning and DMIRE2 Leica CLSM TCS-SP2 confocal laser scanning microscope. The obtained TIFF images were resized and overlays were prepared with Adobe Photoshop. Statistical analysis of the effect of infection on distribution of host cell proteins was carried out using the *chisq.test()* function in R.

### Co-immunoprecipitation

NSDVi-infected cells were harvested with 0.4 ml of a lysis buffer (1% (v/v) Nonidet P-40, 50 mM Tris/Cl pH 7.5, 150 mM NaCl, 2 mM EDTA, 2.5 mM EDTA) containing protease inhibitor cocktail set III (Calbiochem) at a final dilution of 1/200. Lysates were centrifuged at 10,000 g for 30 min at 4C and the supernatants were subjected to immunoextraction with 1 μl of an appropriate antibody and using protein G-agarose beads (Millipore). After a series of washes (2x with 0.2% NP40, 10 mM Tris/Cl pH 7.5, 150 mM NaCl, 2 mM EDTA; 2x with 0.2% NP40, 10 mM Tris/Cl pH 7.5, 500 mM NaCl, 2 mM EDTA; and 1x with 10 mM Tris/Cl pH 7.5), the immunoprecipitated proteins were analysed by sodium dodecyl sulphate polyacrylamide gel electrophoresis (SDS-PAGE) and immunoblotting using specific antibodies.

### Immunoblotting

At the indicated times, infected or transfected cells were harvested and lysed with 100 μl of 1x SDS sample buffer (New England Biolabs). SDS-PAGE and Western blots were carried out as previously described [65].

To analyse secreted proteins, cell were cultured in 12-well plates containing 0.5 ml of growth medium per well. The medium was collected, pre-cleared of cellular debris by centrifugation at 664 g for 10 min at 4°C, and supernatants were mixed with 5x SDS-PAGE loading buffer (4% SDS, 250 mM Tris/Cl pH 6.8, 0.6 mg/ml of bromophenol blue, 7.5 mM DTT, 30% (v/v) glycerol in water). Such prepared protein lysates were analysed by Western blotting as described above.

## Results

### NSDV-infected cells show reduced levels of PDI and ERp57 while the structure of the ER, ERGIC and Golgi remains unchanged

Two isolates of NSDV have been previously described by us [29]: a multiple-times passaged isolate of NSDV from Uganda, which appeared attenuated upon infection of a susceptible animal, and an isolate of GV from India which had been passaged a limited number of times in mouse brain or BHK21 clone 13 cells and which caused haemorrhagic gastroenteritis in sheep upon experimental inoculation. Since NSDV and GV have been shown to be the same virus [9], [10], for simplicity we have referred to them both as NSDV in this paper, NSDVi for the pathogenic and NSDVu for the apathogenic isolate. We focussed on the pathogenic isolate as the best representative of a wild-type virus; this virus replicated reasonably well in Vero cells, although it grew poorly in most of the other cell lines tested [29].

**Figure 1:**
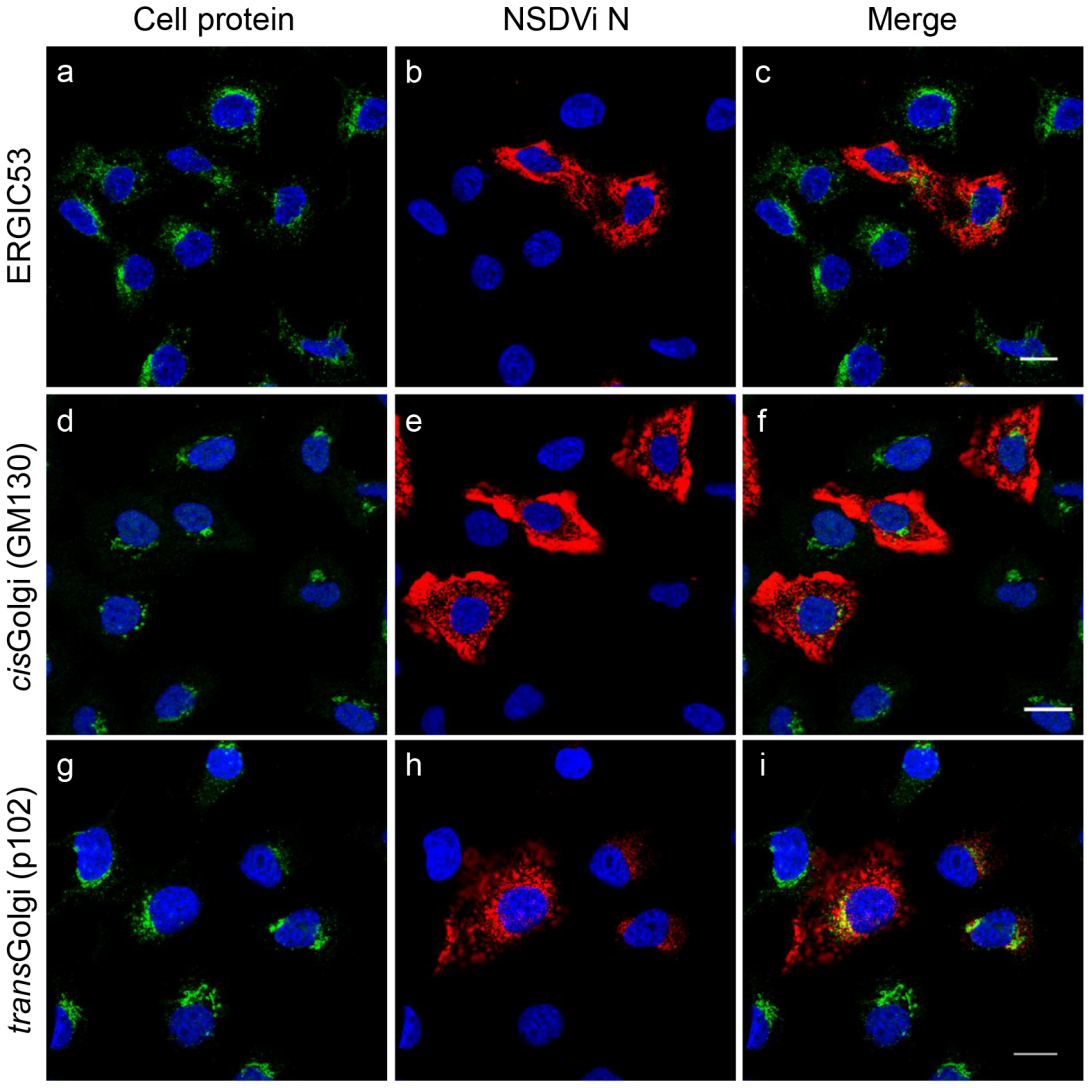
NSDV replication has no obvious effect on the ERGIC and Golgi. Vero cells were infected with the NSDVi isolate at a MOI of 0.3 TCID_50_. After 16 h, cells were fixed using 3% PFA followed by ice-cold methanol. Cells were co-stained with mouse anti-ERGIC53 (ERGIC; a–c), mouse anti-GM130 (*cis*Golgi; d–f) or rat anti-p102 (*trans*Golgi; g–i) and rabbit antiserum against the viral N protein. Proteins were visualised by co-staining with Alexa Fluor 488 goat anti-mouse or anti-rat IgG (green), and Alexa Fluor 568 goat anti-rabbit IgG (red). Nuclei were counterstained using DAPI (blue). Bars correspond to 20 μm.

Vero cells were infected with NSDVi at a low multiplicity of infection (MOI), which allowed for direct comparison of infected and uninfected cells on a single slide and even in one focal view. After overnight infection, cells were fixed and stained with antibodies specific for ERGIC (ERGIC53), *cis*Golgi (GM130) or *trans*Golgi (p102) markers. Infected cells were detected using rabbit polyclonal antiserum against the viral N protein (which recognises both NSDVi and NSDVu isolates), and the distribution of the cellular proteins was compared in infected and uninfected cells by confocal microscopy. Apart from a few infected cells where the N protein appeared to fill up the space occupied by the ERGIC, no obvious changes to the distribution or amount of ERGIC53 was observed (Figure 1.A a–c). Similar results were observed for the *cis* and *trans*Golgi compartments, with no obvious changes to the distribution of GM130 and p102 (which are matrix proteins of the *cis* and *trans*Golgi respectively [63], [66]) when infected cells were compared to uninfected cells (Figure 1.A d–i). NSDV replication did not appear to affect protein levels of these markers, as judged by fluorescence intensity. The same results were obtained for NSDVi-infected A549 cells, and for Vero and A549 cells infected with NSDVu (data not shown). These results indicate that, despite budding into the Golgi, often shedding large numbers of the virus particles [35], NSDV does not appear to alter ERGIC and Golgi structures (at least not sufficiently for the alteration to be detected by confocal microscopy). This suggests that the Golgi and ERGIC remain functional during infection, thereby promoting viral egress. NSDV replication also had no apparent effect on microtubule and actin filament organisation (Figure S1); in addition, several NSDV-infected cells were found undergoing mitosis, showing a well-defined mitotic spindle as observed by α-tubulin staining, indicating that the centrioles likewise remained functional in NSDV-infected cells (Figure S1).

**Figure 2:**
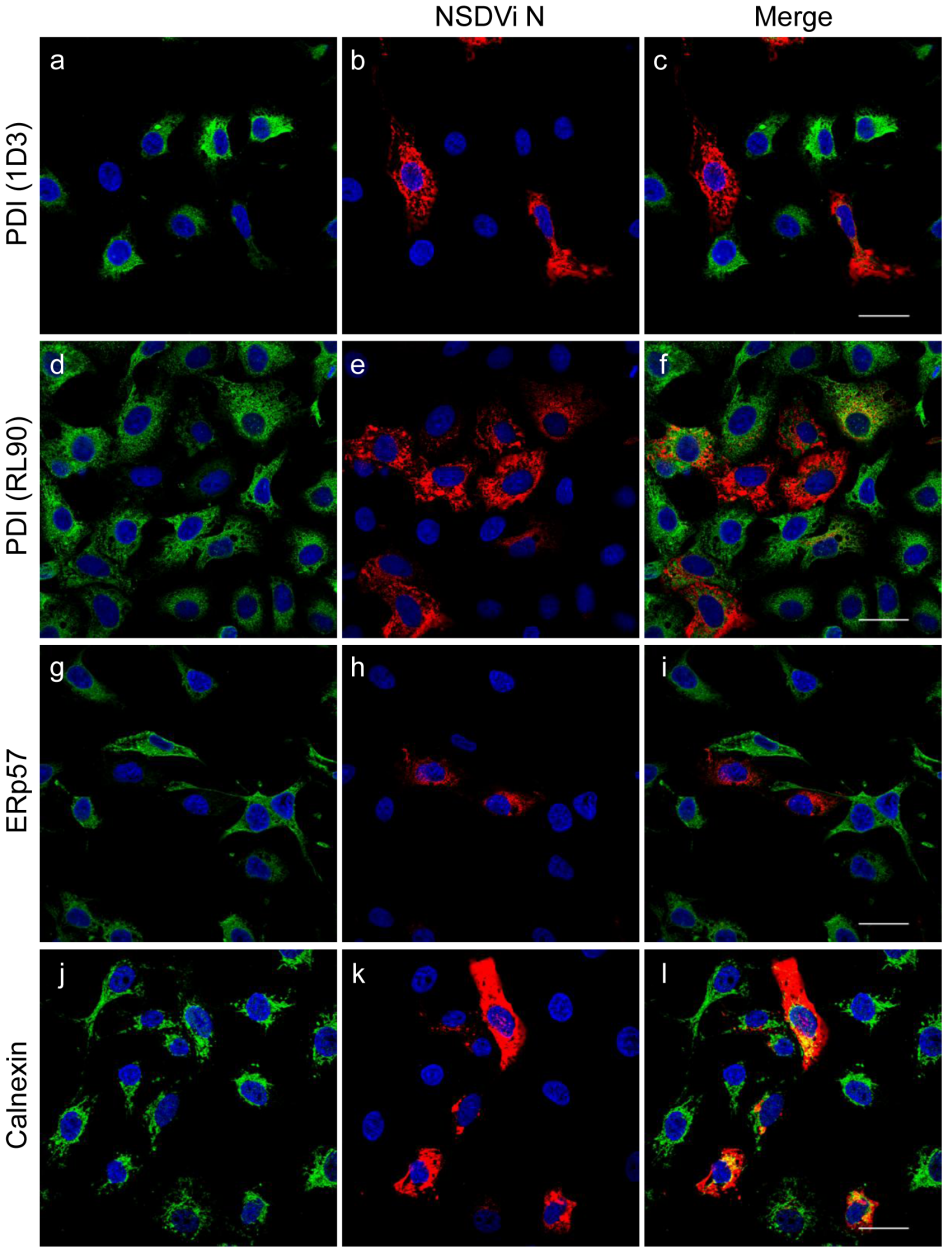
NSDV-infected cells show reduced level of PDI and ERp57 in the ER. The experiment was carried out as for Figure 1, but cells were co-stained with mouse anti-PDI (clone 1D3; a–c), mouse anti-PDI (clone RL90; d–f), rabbit anti-ERp57 (g–i) or mouse anti-calnexin (j–l), and rabbit antiserum against the viral N protein. Proteins were subsequently visualised by co-staining with Alexa Fluor 488 goat anti-mouse IgG (green) and Alexa Fluor 568 goat anti-rabbit IgG (red) (a–f and j–l), except for g–i, where rabbit anti-ERp57 IgGs were labelled with Zenon Alexa Fluor 488 rabbit IgG labelling reagent (green) and rabbit anti-N IgGs were labelled with Zenon Alexa Fluor 594 rabbit IgG labelling reagent (red). Bars correspond to 40 μm.

A different picture was observed when the ER of infected cells was observed. When staining cells with mouse anti-PDI antibody clone 1D3, which targets the C-terminal part of PDI, containing the KDEL retention motif [67], we discovered that in many infected cells the amount of PDI appeared to decrease, even disappearing completely from the ER of about 1 in 3 infected cells (Figure 2 a–c). To exclude the possibility that the viral glycoproteins mask the epitope recognised by the antibody clone 1D3, the experiment was repeated using mouse anti-PDI antibody clone RL90, which targets a different PDI epitope from that recognised by 1D3, since it inhibits the activity of PDI *in vitro* [68] and therefore recognises a region close to the enzymatic site of the protein. Once again, many infected cells showed a reduced amount of PDI in the ER (Figure 2 d–f), confirming that, in NSDV-infected cells, PDI shows a different staining pattern than in uninfected cells, and that many infected cells even appear to lose PDI entirely. The distribution of ERp57, another soluble chaperone of the PDI-like family, was also studied in NSDV-infected cells; again many infected cells showed a similar redistribution or complete loss of ERp57 from the ER (Figure 2 g–i), indicating that other ER-lumenal chaperones/proteins might also be affected. The fraction of infected cells in which PDI distribution was affected was quantified visually. The amount of PDI in the ER of 150 infected and uninfected cells was investigated and approximately 33% of infected cells (53/150) were found to show either a drastically reduced level or total loss of intracellular PDI, while only 1 uninfected cell was seen with low/absent PDI in the ER (χ^2^ = 58.7, p<0.00001).

In order to establish whether NSDV replication affects the structure of the entire ER or just the distribution of specific proteins, we looked at the distribution of calnexin, an ER-membrane chaperone, in infected and uninfected cells. No change to the cellular distribution of calnexin was observed (Figure 2 j–l), indicating that, despite the disappearance of PDI and ERp57, the overall structure of the ER remains unchanged in NSDV-infected cells.

### PDI and ERp57 are not degraded in NSDV-infected cells

**Figure 3:**
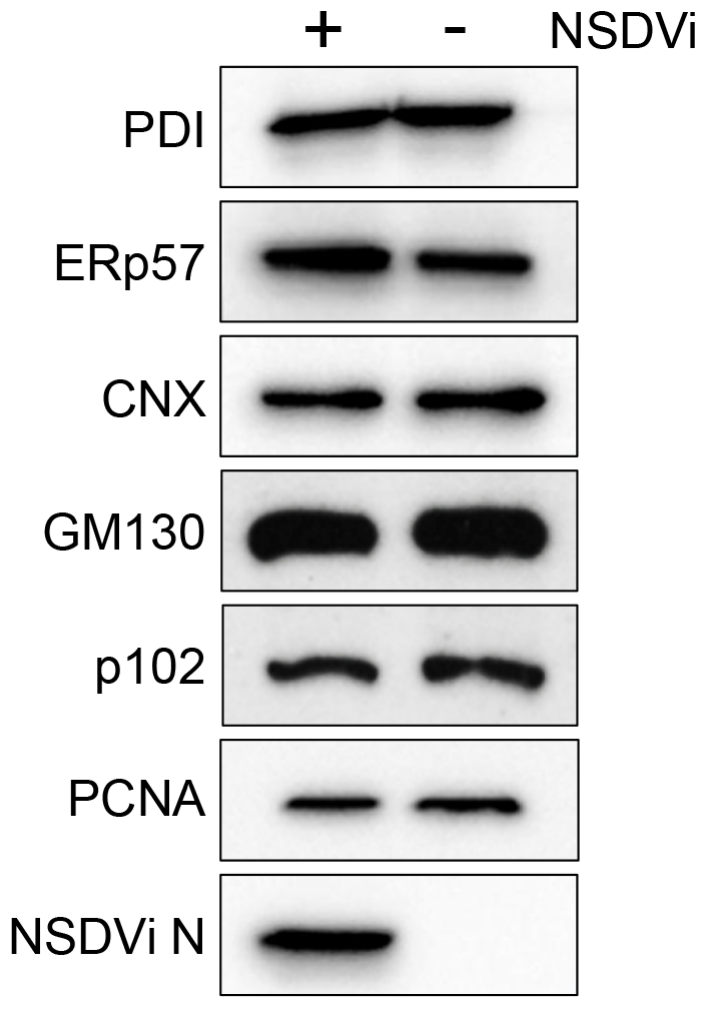
PDI and ERp57 appear not to be degraded in NSDV-infected cells. Vero cells were infected with the NSDVi isolate at a MOI of 5 TCID_50_ or left uninfected. After 16 h, cells were harvested by lysis and proteins separated on an acrylamide SDS-PAGE gel; proteins were detected by Western blot using antibodies specific to PDI (clone RL90), ERp57, calnexin (CNX), *cis* Golgi (GM130), *trans* Golgi (p102), PCNA and the NSDV N protein.

To examine whether PDI and ERp57 are selectively degraded during infection, Vero cells were infected with NSDVi at a high MOI such that all cells were infected, and total cellular levels of PDI and ERp57 in these cells were analysed by Western blotting. For comparison, we also determined the relative levels of calnexin, GM130 and p102 (proteins which showed no effect of infection on their distribution in individual cells by immunofluorescence); proliferating cell nuclear antigen (PCNA) used as a loading control. Surprisingly, given the confocal results, NSDVi infection appeared to have no effect on the total PDI and ERp57 protein levels (Figure 3). In the case of very abundant proteins such as PDI, which represents around 1% of total cellular proteins (reviewed in [69]), a small difference in protein level might not be detected by Western blotting; however, during NSDV infection, 33% of the infected cells showed major or total loss of PDI (as estimated using immunofluorescence), therefore some change in the total PDI level would be expected to be seen if this protein was degraded in the infected cells. These data indicate that, although PDI and ERp57 disappear from the ER of infected cells, this is not a reflection of overall loss from the cell such as through degradation.

### PDI and ERp57 appear at the surface of many NSDV-infected cells and are secreted from these cells

**Figure 4:**
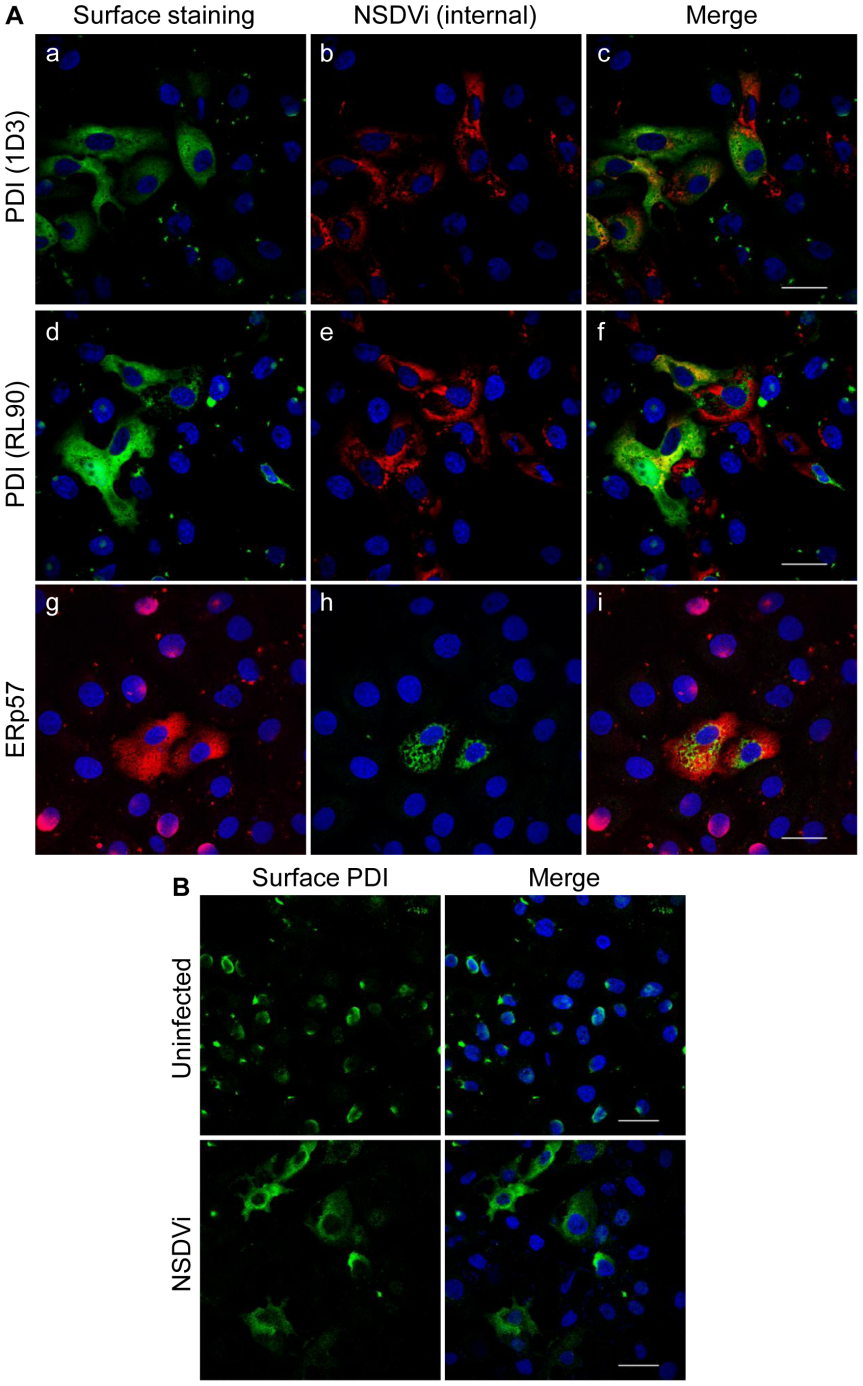
PDI and ERp57 appear at the surface of NSDV-infected cells. **A.** Samples were prepared as for Figure 1 except that, after fixing with 3% PFA, cells were labelled with mouse anti-PDI (clone 1D3; a–c), mouse anti-PDI (clone RL90; d–f) or rabbit anti-ERp57 (g–i). Then cells were again fixed with 3% PFA, opened with ice-cold methanol, and stained with rabbit antiserum against the viral N protein (a–f) or with mouse anti-PreGn antibody (g–i). Proteins were visualised by co-staining with Alexa Fluor 488 goat anti-mouse IgG (green) and Alexa Fluor 568 goat anti-rabbit IgG (red). **B.** Vero cells were infected with the NSDVi isolate at a MOI of 6 TCID_50_ or left uninfected. After 16 h, cells were fixed using 3% PFA and left non-permeabilised. Cells were incubated with mouse anti-PDI (clone 1D3) antibody followed by Alexa Fluor 488 goat anti-mouse IgG (green). Nuclei were counterstained using DAPI (blue). Bars correspond to 40 μm.

The absence of PDI and ERp57 degradation suggested that, although these proteins disappear from the ER, they somehow remain associated with the NSDV-infected cells. One possibility was that these proteins are moving to the cell surface in infected cells, but are lost during fixation and washing, since PDI is known to be only weakly bound to the cell surface via non-covalent interactions with other molecules present at the cell surface [70]. This possibility was investigated using careful surface labelling of lightly fixed cells and confocal microscopy. To detect PDI and ERp57 at the surface, Vero cells infected with NSDVi at a low MOI were fixed and then incubated with anti-PDI or anti-ERp57 antibody without permeabilization. After washing away unbound antibodies, cells were again fixed with 3% PFA to ensure that any PDI/ERp57 and bound antibodies remained attached to the cell surface throughout the staining procedure. To detect NSDV proteins, cells were then permeabilised with ice-cold methanol and incubated with rabbit anti-NSDV N protein serum or with mouse anti-NSDVi PreGn (a monoclonal which recognises a sequence near the amino-terminus of the NSDVi PreGn glycoprotein). We observed that many NSDV-infected cells showed significant amounts of cell surface PDI and ERp57 (Figure 4.A). Although small amounts of PDI and ERp57 could be observed at the surface of even uninfected cells, NSDV infection led to a large increase in the amount of these proteins at the surface. Approximately 1/3^rd^ of infected cells (calculated as a fraction (51/150) of viral protein-positive cells with increased surface PDI/ERp57 calculated over several focal views) showed this high level of surface PDI/ERp57, and only 1/150 uninfected cells showed increased surface PDI/ERp57 (χ^2^ = 55.8, p<0.00001). We did observe a small fraction of cells the membranes of which were permeabilised by the initial fixation, leading to labelling of internal PDI or ERp57; however, surface PDI/ERp57 staining showed a completely different pattern to the resultant intracellular (ER) staining.

To ensure that the apparent surface PDI is not an artefact of the multi-step labelling procedure and that anti-PDI antibody does not re-associate with an internal epitope after opening of cells with methanol, we infected cells at a high MOI and repeated the surface labelling of non-permeabilised cells using anti-PDI antibody as previously, but omitting the subsequent permeabilization step and staining for viral protein. Enhanced surface PDI was detected in batches of infected but not in uninfected cells (Figure 4.B), confirming that surface PDI detected in NSDV-infected cells is not an artefact of the staining procedure. The disappearance of PDI/ERp57 from the ER and their appearance at the cell surface in infected cells suggests that NSDV replication and/or assembly induces movement of these proteins from the ER to the cell surface.

**Figure 5:**
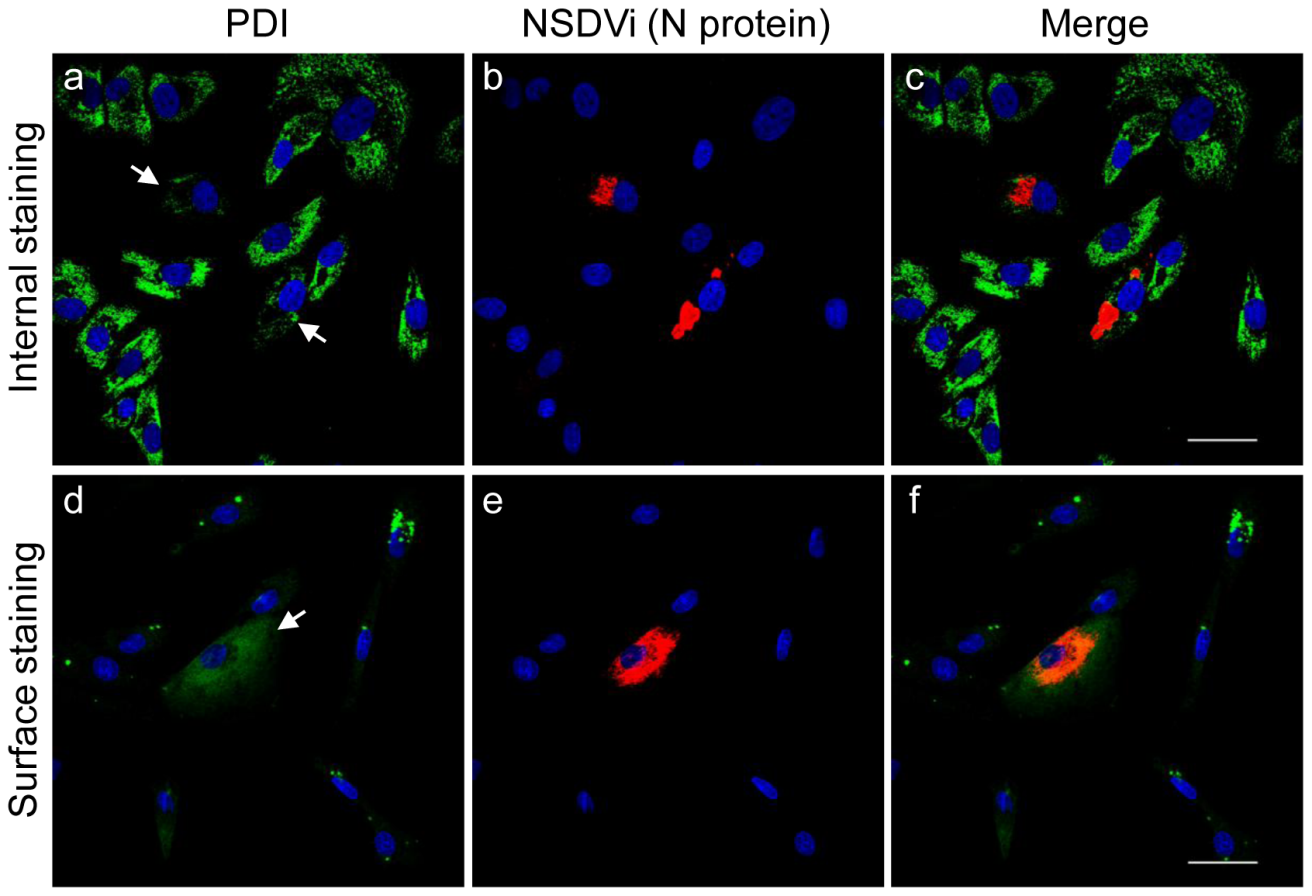
Effect of NSDV infection on the distribution of PDI in a caprine endothelial cell line (CJE). CJE cells (a caprine endothelial cell line) were infected with the NSDVi isolate at a MOI of 6 TCID_50_. After 72 h, cells were fixed with 3% PFA followed by ice cold methanol (a–c) or with 3% PFA only (d–f). Cells were co-stained with mouse anti-PDI (clone 1D3) antibody and rabbit antiserum against the viral N protein (a–c), or stained only with mouse anti-PDI antibody, then again fixed with 3% PFA followed by treatment with ice-cold methanol and stained with rabbit antiserum against the N protein (d–f). Proteins were visualised by Alexa Fluor 488 goat anti-mouse IgG (green) and Alexa Fluor 568 goat anti-rabbit IgG (red). Nuclei were counterstained using DAPI (blue). Bars correspond to 40 μm; arrows in “a” and “d” indicate infected cells.

The effect of NSDV replication on PDI distribution in the cell was also tested in other cell types. While NSDVi grows relatively poorly in a caprine endothelial cell line (CJE), it still induced redistribution of PDI from the ER to the cell surface in these cells (Figure 5). Similar changes to the distribution of PDI upon infection with NSDV (either NSDVi or NSDVu) were also observed in A549 cells and in a bovine foetal endothelial cell line (BFA) (Figure S2). This confirmed that redistribution of PDI during NSDV infection is not Vero cell-specific, nor is it specific to a pathogenic or apathogenic virus type.

**Figure 6:**
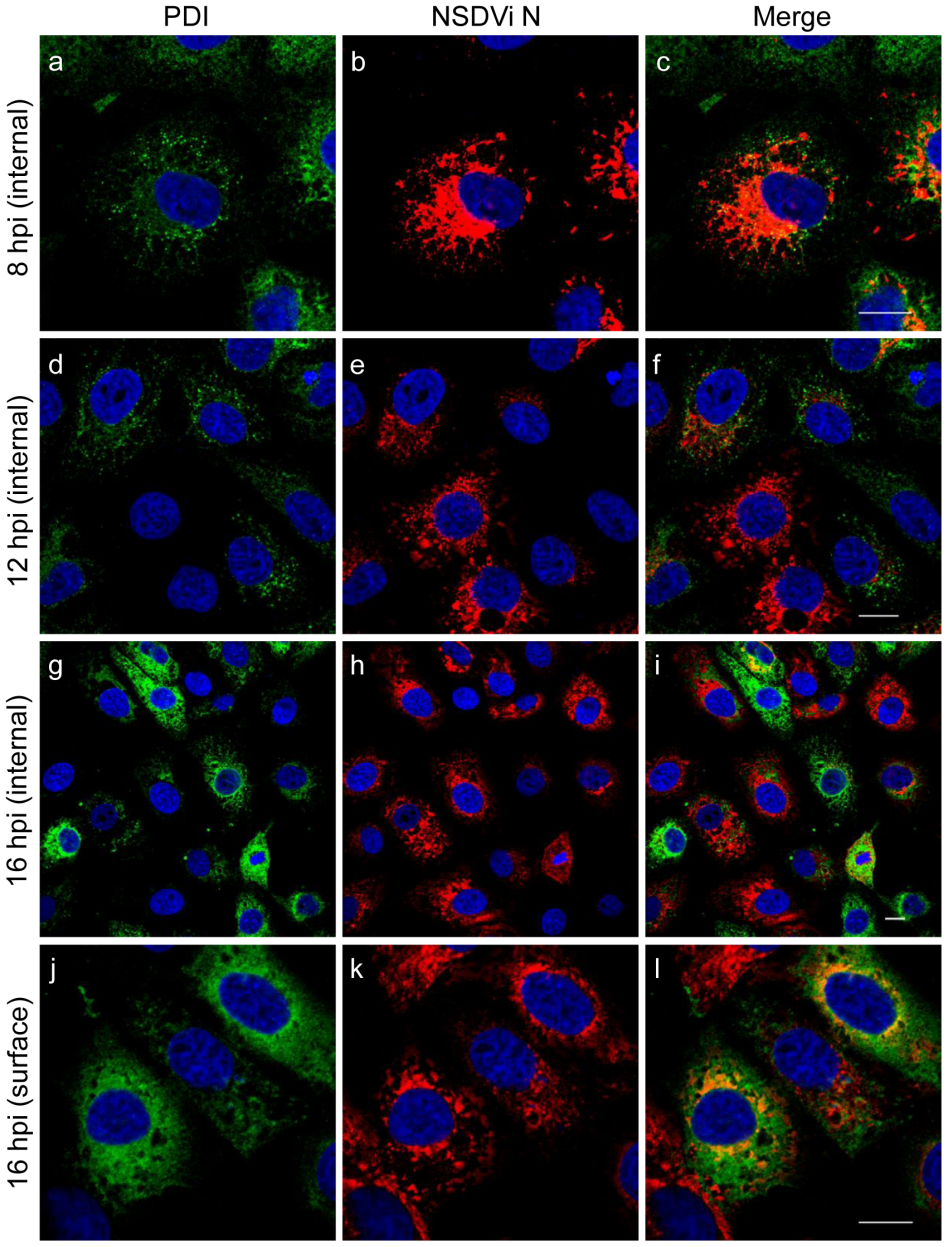
Time course of changes to PDI in NSDV-infected cells. Vero cells were infected with the NSDVi isolate at a MOI of 6 TCID_50_ and fixed at 8, 12 and 16 hpi. For internal staining (a–i) cells were fixed using 3% PFA followed by ice cold methanol. Cells were co-stained with mouse anti-PDI (clone 1D3) antibody and rabbit antiserum against the viral N protein. For surface PDI staining (j–l), cells were fixed with 3% PFA followed by staining with mouse anti-PDI (clone 1D3) antibody. Then cells were again fixed with 3% PFA, opened with ice-cold methanol, and virus was detected by rabbit antiserum against the N protein. Proteins were visualised with Alexa Fluor 488 goat anti-mouse IgG (green) and Alexa Fluor 568 goat anti-rabbit IgG (red). Nuclei were counterstained using DAPI (blue). Bars correspond to 16 μm.

NSDVi-infected cells were studied at different times post infection to determine when during infection changes to the distribution of PDI appear. Vero cells were infected at high MOI and fixed at 0, 1, 2, 4, 8, 12 and 16 hours post infection (hpi), after which PDI was labelled in the ER or at the surface. The first changes to PDI distribution could be observed at 8 hpi, when a few cells showed a reduced amount of PDI in the ER (observed visually as a clearly weaker fluorescence signal when compared to uninfected cells) and vesicle-like structures containing PDI could be noticed (Figure 6 a–c). At 12 hpi, several cells which had completely lost detectable PDI from the ER were observed (Figure 6 d–f) and at 16 hpi many cells lacking PDI were found (Figure 6 g–i). The first surface PDI was only seen at 16 hpi (Figure 6 j–l); however, it is possible that a small increase of surface PDI would be missed at earlier time points due to the relatively low sensitivity of confocal microscopy. Similar results were obtained for ERp57 (Figure S3). Interestingly, despite synchronising infection by washing away unbound virus 1 h post inoculation, it was observed that infection had not progressed to the same degree in all infected cells, so that, at 8 hpi, only a few cells showed detectable viral N protein, while at 12 hpi, at which time progeny virus can be detected [29], cells with very different amounts of N were observed (Figure 6). This apparent variation in the extent of virus replication in individual cells from the same sample may be due to variation in the virions themselves (e.g. individual virus particles having different numbers of copies of each segment) or due to the virus replicating with different rates in different cells, possibly due to some effect of cell cycle, as the cell cycle of the cells used in this experiment was not synchronised.

We also observed that some NSDVi-infected cells, despite containing large amount of N protein, showed very little or no PreGn (Figure S4), which could explain why not all cells judged to be infected because of the presence of viral N protein had lost PDI from their ER or showed surface PDI expression. Differential expression of the N and PreGn proteins in NSDV-infected cells may reflect the apparent non-equimolar amount of the three viral RNA segments found previously in number of bunyaviruses [40], [71]–[73].

**Figure 7:**
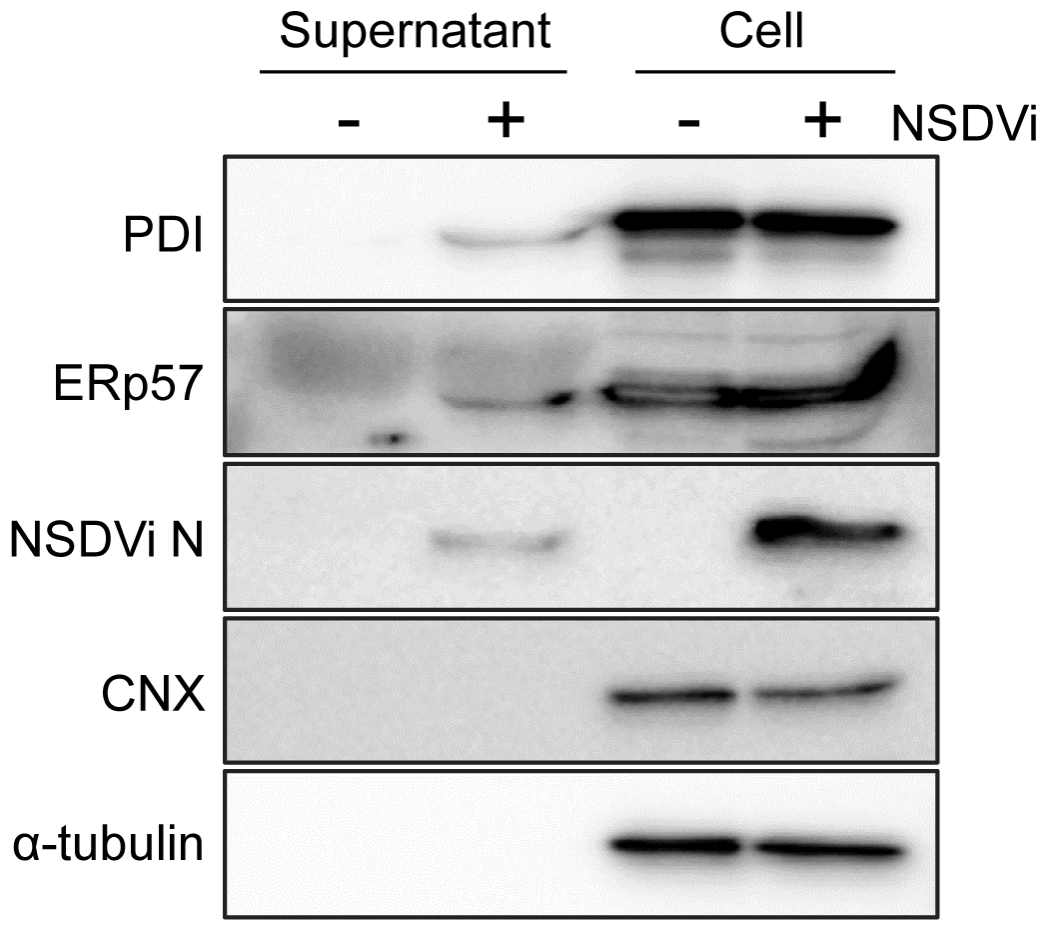
The replication of NSDV induces secretion of PDI and ERp57 from infected cells. Vero cells were infected with the NSDVi isolate at a MOI of 0.1 TCID_50_ or left uninfected. After 48 h, supernatants and cells were harvested; proteins were separated on acrylamide SDS-PAGE gels and cellular and viral proteins were detected by Western blotting using specific antibodies to PDI (clone RL90), ERp57, the viral N protein, calnexin (CNX) or α-tubulin.

Since PDI is a soluble protein which is thought to be retained at the cell surface through non-covalent interactions [70], and the secretion of PDI has been observed from a number of cell types [70], [74], we investigated whether PDI and ERp57 not only appear at the surface of NSDV-infected cells but are also secreted from these cells. Vero cells were again infected with NSDVi and culture supernatants (i.e. the cell medium) and the cells themselves were harvested, and proteins from supernatants and/or cell lysates were analysed by Western blotting using specific antibodies. Before harvesting, cells were checked by light microscopy for the appearance of any cytopathic effect (CPE), to ensure that only the medium from cells which appeared healthy was used in this study. After 48 h, in cells infected with NSDVi at a low MOI, no CPE was observed in the culture, but a clear increase in levels of PDI and ERp57 was detected in the medium. These proteins were found in the medium from infected cells but not from uninfected cells (Figure 7), while cellular proteins such as tubulin or calnexin were not found in the medium (Figure 7), indicating that the PDI, ERp57 and NSDV N protein were not simply coming from ruptured cells.

### Role of specific glycoproteins in the redistribution of PDI in NSDV-infected cells

**Figure 8:**
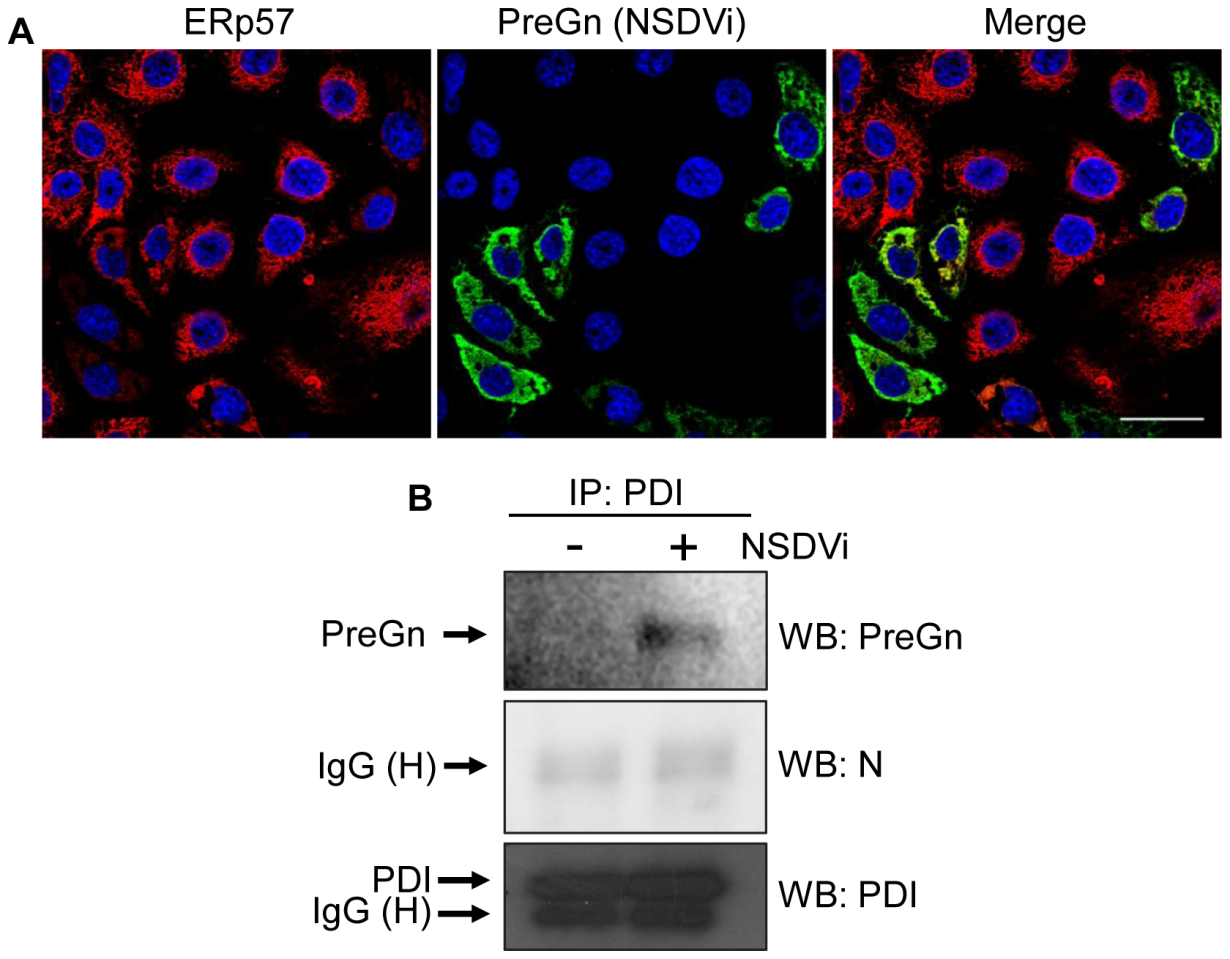
Viral glycoprotein association with PDI and ERp57. **A.** Samples were prepared as for Figure 1 except that cells were co-stained with rabbit anti-ERp57 and mouse anti-PreGn antibodies followed by co-staining with Alexa Fluor 488 goat anti-mouse IgG (green) and Alexa Fluor 568 goat anti-rabbit IgG (red). Bar corresponds to 40 μm. **B.** Vero cells were infected with the NSDVi isolate at a MOI of 0.1 TCID_50_ or left uninfected. After 48 h, cells were harvested and proteins were immunoprecipitated (IP) from cell lysates using mouse anti-PDI (clone RL90) antibody and protein G-agarose beads. Proteins were separated on acrylamide SDS-PAGE gels and detected by Western blotting (WB) using anti-PreGn, anti-PDI or anti-N antibodies.

The NSDV glycoproteins mature in the ER and Golgi and their synthesis, like that of most glycoproteins, will involve the ER chaperones. It is therefore possible that the viral glycoproteins may be involved in the redistribution of PDI/ERp57 in infected cells. In order to visualise this directly, we labelled infected cells with rabbit anti-ERp57 and mouse anti-PreGn. It was observed that ERp57 is redistributed in infected cells so that it is essentially all in the specific areas occupied by viral glycoproteins (Figure 8.A), suggesting that these chaperones may be recruited to the parts of the ER involved in synthesising the viral glycoproteins. Although we could not carry out a similar study of PDI/PreGn colocalisation (no good rabbit anti-PDI was found), a similar association of PDI and viral glycoprotein is strongly suggested by studies in which we lysed infected cells and immunoextracted the lysates using mouse anti-PDI (clone RL90) antibody. We found PreGn, but not N, among proteins co-precipitated by anti-PDI antibody from infected cells (Figure 8.B), indicating that PDI appears to interact, directly or indirectly, with PreGn and possibly other of the viral glycoproteins in NSDV-infected cells (Figure 8.B) (note that reverse immunoprecipitation with anti-PreGn was not possible since the monoclonal antibody in question appears to recognise only denatured PreGn).

We constructed plasmids expressing NSDV glycoproteins PreGn, NS_M_ or PreGc to determine which glycoprotein(s) were responsible for the redistribution of PDI/ERp57. *In silico* analysis of the protein sequence encoded by the NSDV M segment suggested that the NSDV glycoproteins have a similar organisational arrangement to that described for CCHFV [50], although with some differences in the exact lengths of the different proteins. Transmembrane (TM) regions and signalase cleavage sites were predicted using TMHMM Server v. 2.0 and SignalP 4.0 Server [75] respectively; six hydrophobic regions TM_0_ (N-terminal), TM_1_ (631-653 aa), TM_2_ (751-773 aa), TM_3_ (786-803 aa), TM_4_ (907-928 aa) and TM_5_ (1524-1546 aa) were predicted with signalase cleavage sites just after the hydrophobic sequences of TM_0_ (19↓20 aa), TM_2_ (770↓771 aa) and TM_4_ (927↓928 aa). Based on the NSDV M topology prediction, plasmids encoding PreGn, NS_M_ and PreGc (corresponding respectively to aa 1–770, 750–927 and 907–1624 of the NSDVi M open reading frame) were constructed in which each protein was in frame with a carboxy-terminal V5 epitope tag. Expression of these constructs was tested in Vero cells by Western blotting and immunofluorescence to ensure that proteins of the expected size and localising to the expected compartments were expressed. The plasmid-expressed PreGn-V5 was found in the ER and *cis*Golgi, while the PreGc-V5 was found only in the ER, with no detectable colocalisation with the *cis*Golgi marker (Figure S5 a–f). Some of the plasmid-expressed NS_M_-V5 protein colocalised with the *cis*Golgi marker, however NS_M_-V5 also appeared in other compartments of the secretory pathway, one of which could be ERGIC (Figure S5 g–i).

**Figure 9:**
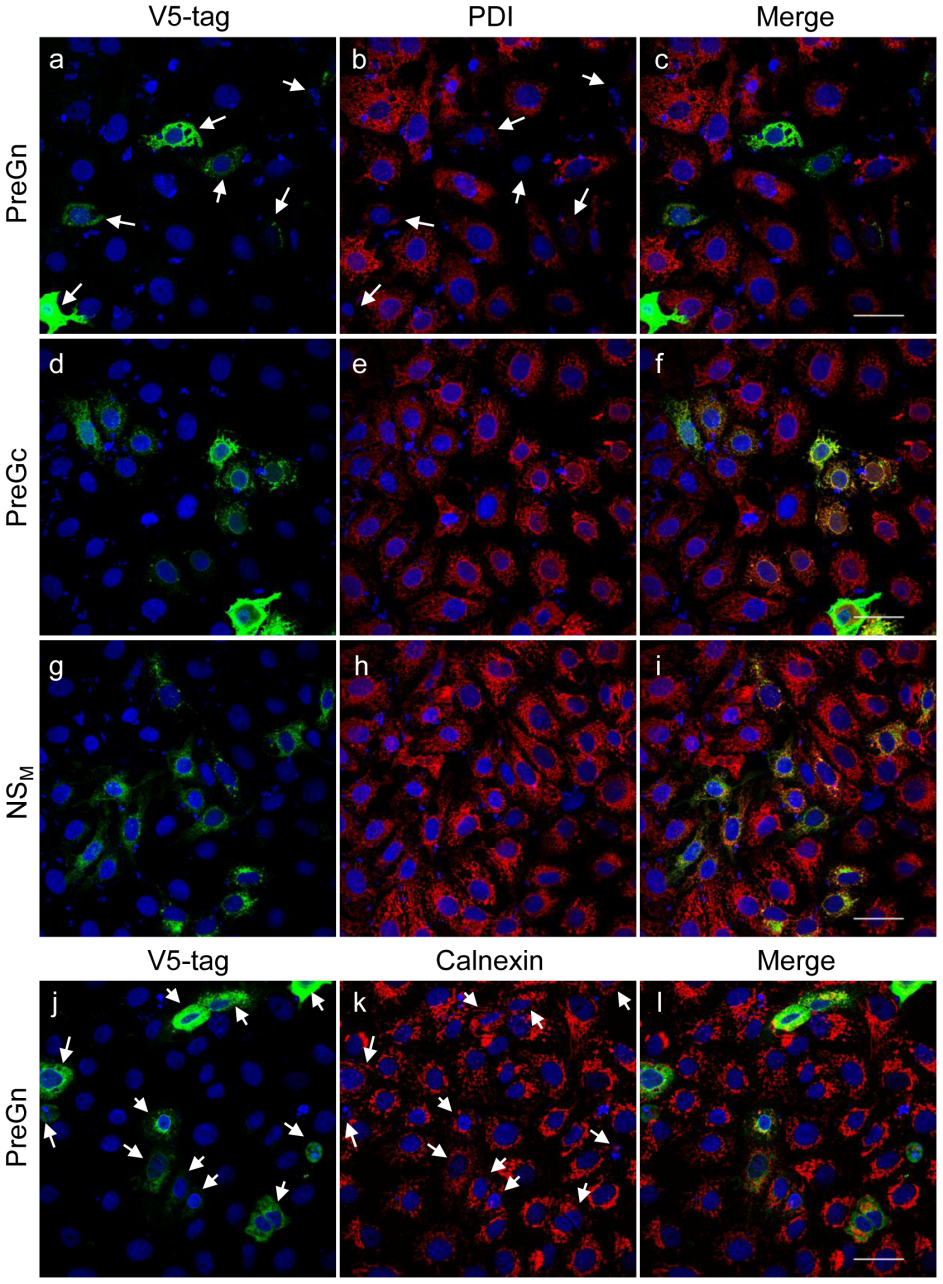
The effect of NSDV glycoprotein expression on PDI. Vero cells were transfected with 1 μg of pCAGGs_MCSII_PreGn_V5 (a–c and j–l), pCAGGs_MCSII_PreGc_V5 (d–f) or pCAGGs_MCSII_NS_M__V5 (g–i). After 24 h, cells were fixed with 3% PFA followed by ice-cold methanol, and were stained using mouse anti-PDI (a–i) or anti-calnexin (j–l) antibodies followed by Alexa Fluor 568 goat anti-mouse IgG (red). Plasmid-expressed proteins were visualised with mouse anti-V5 antibody conjugated to Alexa Fluor 488 (green). Nuclei were counterstained using DAPI (blue). Bars correspond to 40 μm. Arrows in a–b and j–k indicate cells expressing PreGn.

Vero cells were transfected with the PreGn-V5, PreGc-V5 and NS_M_-V5 expression constructs and their effect on PDI distribution in transfected cells was investigated by confocal microscopy. Cells which expressed PreGn-V5 lost PDI from the ER (Figure 9 a–c), while expression of PreGc-V5 or NS_M_-V5 had no apparent effect on the PDI distribution (Figure 9 d–i), suggesting that it is the (Pre)Gn but not (Pre)Gc or NS_M_ which is primarily responsible for the translocation of PDI, and possibly ERp57, to the cell surface. Although in some cells over-accumulation of PreGn-V5 appeared to change the morphology of the ER, the cells which showed only moderate or low levels of PreGn-V5 in the ER also lost PDI from this compartment. In cells in which PreGn-V5 was found only in the Golgi (i.e. cells which expressed PreGn at a very low level) no change to PDI was observed, suggesting that PreGn needs to accumulate in the ER, even at low levels, in order to affect the distribution of PDI. PreGn-V5 had no effect on the distribution of calnexin (Figure 9 j–l), indicating that the PreGn-V5 does not affect the overall ER structure when expressed at moderate levels, similarly to what was observed in NSDV-infected cells. As expected, expression of the viral N protein or the N-terminal half of the viral L protein (La) [62] had no effect on PDI distribution (Figure S6).

Taken together, these data suggest that NSDV infection causes the re-localisation of ER soluble proteins, notably PDI and ERp57, to the cell surface and ultimately the medium surrounding the infected cell, and this re-localisation appears to be due to association of these proteins with the NSDV (Pre)Gn glycoprotein.

## Discussion

While both NSDV and CCHFV are nairoviruses which cause a highly pathogenic haemorrhagic disease in their natural hosts, little is known about the mechanism of pathogenesis of these viruses; even the primary cellular targets are currently unknown. To better understand the replication of nairoviruses we have studied NSDV and its effects on the cellular compartments in infected cells, particularly concentrating on the host cell secretory pathway, which is used by nairoviruses for budding and egress of newly generated virions.

Despite a high throughput of viral glycoproteins processed through the secretory compartments, and viral budding in the Golgi, the structure of the ER and post-ER compartments appeared to be unaffected in NSDV-infected cells when investigated using immunofluorescence. While what appear to be swollen Golgi cisternae and vacuolisation of the cytoplasm has been observed by electron microscopy in NSDV-infected cells [35], it is possible that changes to the Golgi observed by electron microscopy are not extensive enough to be detected by confocal microscopy, or are not reflected in changes to the distribution of protein markers of those compartments. Our findings agree with the absence of changes to the Golgi observed in cells infected with CCHFV or Hantaan virus (HTNV; a hantavirus) [76], [77]. In contrast, Bunyamwera virus (BUNV; an orthobunyavirus)-infected cells showed a condensed phenotype of the *cis* and *trans*Golgi [78]–[80], indicating that different bunyaviruses may have different effects on the Golgi. While the ERGIC has been proposed as the first site of budding for phleboviruses, orthobunyaviruses and hantaviruses [76], [79]–[83], no specific accumulation of the NSDV N colocalising with the ERGIC marker was observed in our studies. Although some overlapping of N and ERGIC53 signal was noticed in some of the infected cells, such apparent colocalisation appeared to be due to the accumulation of very high levels of N protein in those cells.

While the structure of the ER remains unchanged, NSDV infection led to the redistribution of the soluble oxidoreductases PDI and ERp57 from the ER to the cell surface, and ultimately to the extracellular space (i.e. cell medium). Redistribution of PDI has been observed also during replication of other viruses. For instance, PDI was redistributed from the peripheral ER to perinuclear membranous viral factories induced by Kunjin virus (KUNV; genus *Flavivirus*) [84]. It is currently unknown whether KUNV selectively redistributes PDI or also affects other ER proteins; since the KUNV glycoproteins E and M mature in the ER [85] and KUNV generates its viral factories by remodelling both the ER and *trans*Golgi [84], it is possible that in KUNV-infected cells the normal retrieval system of ER proteins may not work. Interestingly, despite changes to the ER, no major effect on the Golgi structure was found by immunofluorescence in KUNV-infected cells [84]. Another flavivirus, Dengue virus (DENV) (which, like NSDV, can cause a haemorrhagic fever), increased surface levels of PDI in infected endothelial cells [86]. As in our observations on NSDV-infected cells, total PDI levels were not affected in DENV-infected cells [86]; at present it is unknown whether DENV replication affects the distribution of intracellular PDI. Although some redistribution of PDI was found in BUNV-infected Vero cells, these changes most probably result from fragmentation of the ER induced by the virus and this effect on PDI appears to be Vero–specific, since these changes have not been observed in BUNV-infected BHK21 cells [79]. No effect on PDI was detected in HTNV-infected cells [76].

Redistribution of soluble ER proteins PDI, ERp57 and colligin (a collagen-binding protein) has also been found in cells infected with African swine fever virus (ASFV) [87], [88], a DNA virus which belongs to the family *Asfarviridae* and which causes a severe haemorrhagic disease in domestic pigs. In ASFV-infected cells, PDI, ERp57 and colligin were redistributed from the ER to the ERGIC (which contained the ASFV protein pXP124L) or even entirely disappeared from the ER, while the structure of the ER was not affected [87]. Total cellular levels of PDI, ERp57, and colligin were not affected in ASFV-infected cells and redistribution of these ER soluble proteins was also observed in cells containing plasmid-expressed viral protein pXP124L, suggesting that these ER proteins are not physically redistributed due to wrapping of the ASFV progeny virions in the ER but due to a specific effect of pXP124L. Interestingly, all these very distinct viruses (a negative-strand RNA virus, positive-strand RNA viruses or a DNA virus) use membranes of the secretory pathway to assemble their virions [35], [85], [89], [90], and NSDV, DENV and ASFV all cause haemorrhagic disease [2], [91], [92].

The underlying mechanism for the redistribution of these soluble ER proteins in NSDV infected cells remains to be determined. PDI and ERp57, which contain an ER retention motif at their carboxy-terminus, would normally be bound to KDEL receptors in the ERGIC or *cis*Golgi and recycled back to the ER, although it is known that this process is not totally efficient, and PDI at least has been observed to be released from some cells even under normal conditions [70], [74]. What is more, its release is stimulated in activated endothelial cells [93], [94]. As might be expected due to the generation of large amounts of viral glycoproteins in the ER, NSDV induces the unfolded protein response (UPR) in infected cells (unpublished observation); however, it has been reported that the UPR appeared to diminish PDI secretion [74], so it is unlikely that virus-induced UPR is the cause of the PDI redistribution observed. None of the predicted glycoproteins of NSDV have a C-terminal sequence recognised by KDEL receptors, so the viral proteins cannot be competing with the host cell proteins for KDEL receptors, unlike the case of ASFV pXP124L, the effects of which on ER proteins is dependent on its own C-terminal KDEL motif [87].

Interaction of ER chaperones (e.g. PDI, BiP, calnexin and/or calreticulin) with viral glycoproteins during their folding in the ER would be expected and has been shown for HTNV and UUKV [95]–[97]. It remains to be determined why the PDI is not recycled from the ERGIC or *cis*Golgi in this case, and why only the PreGn protein appears to be involved in PDI distribution and not PreGc. It is possible that bound PreGn makes PDI not available for KDEL receptor. While we have been unable to detect a separate mucin-like protein/GP38 protein, such as that cleaved from the CCHFV PreGn protein by an SKI-1-like protease in the *cis*Golgi [57], [58], in NSDV-infected cells, it may be that final folding of the NSDV (Pre)Gn occurs late in the exit pathway of the virion so that PDI is still required for protein maturation, even after virion assembly. Further maturation of newly synthesised virions in post-*trans*Golgi network has been observed in BUNV-infected cells [79], [80], supporting the possibility that PDI might play a role in late maturation of NSDV. Late maturation, or even virion-bound PDI, may be required to prevent fusion of the viral membrane with the membrane of the secretory compartments during virus egress. Fusion of the CCHFV virion with the endosomal membrane is induced at pH 5.5 to 6.0 [98], which is within the range of pH observed in the lumen of post-Golgi compartments (reviewed in [99]), so a system which prevented fusion of newly synthesised virions with intracellular membranes prior to virus egress would be of benefit.

An alternative is that the virus benefits by inducing PDI release from cells because the PDI is required for virus entry. Extracellular PDI, with its potential for rearranging disulphide bonds within viral glycoproteins, is thought to aid during entry of several viruses [86], [100]–[105]; free thiol groups in the F fusion protein of Newcastle disease virus or the gp120 subunit of the human immunodeficiency virus Env glycoprotein are critical for virus entry and their presence is thought to be mediated by cell surface PDI [100]–[105]. Recent data suggest that free thiol groups at the surface of hantaviruses might also play a role in infectivity of these viruses [106].

Whether NSDV derives a selectable advantage from the redistribution of PDI and ERp57 in infected cells or this redistribution is a bystander effect of the NSDV infection, extracellular PDI may contribute to the pathogenicity observed during NSDV infection. Extracellular PDI can still reduce, oxidise and rearrange disulphide bonds and therefore activate molecules with which it interacts at the cell surface or in the extracellular space (reviewed in [107], [108]). Extracellular PDI has been shown to activate β1 and β3 subunit-containing integrins on endothelial cells and platelets [86], [93], [109]. Integrins are known to take part in the cell-cell interactions promoting cell migration and vasodilation (reviewed in [110]), processes which are enhanced during viral haemorrhagic fevers and which contribute to the haemorrhagic nature of the pathogenesis (reviewed in [111]) e.g. DENV-induced translocation of PDI to the cells surface has been shown to activate integrins on infected endothelial cells [86]. Secreted PDI could also activate integrins at the surface of various uninfected cells, including endothelial cells and platelets, leading to increased vasodilatation and coagulopathy. Extracellular PDI has been shown to activate tissue factor (TF) [112], inducing intravascular coagulation, which may subsequently lead to disseminated intravascular coagulation (DIC) through depletion of platelets and clotting factors, resulting in haemorrhaging [113]–[116]; DIC has been observed in many CCHF patients [31]–[33], [117].

Finally, since ERp57 (and probably PDI) takes part in the folding of the major histocompatibility complex (MHC) class I and therefore assists in facilitating immune surveillance and elimination of virus-infected cells [118], [119], [120,] viruses which translocate these ER chaperones from the ER may avoid adaptive immune responses, although down-regulation of MHC class I has not yet been described for nairovirus-infected cells.

In this study we have demonstrated that NSDV induces the redistribution of soluble ER oxidoreductases, specifically PDI and ERp57, in infected cells, and that the viral PreGn glycoprotein appears to be involved in this process. Redistribution of PDI from the ER of NSDV-infected cells to the cell surface and/or the extracellular environment may play a crucial role in the pathogenicity of NSDV. PDI may enhance virus infection or may contribute to the observed haemorrhagic outcome of the infection, although the exact importance of extracellular PDI and ERp57 in natural infection remains to be determined. In recent years, PDI has become a popular potential drug target for treatment of thrombosis, neurodegenerative diseases and HIV infection [69], [121], [122] and it appears that PDI could also act as a target for treatment of at least some viral haemorrhagic fevers [86]. While a wide range of applications makes PDI inhibitors attractive agents, extracellular PDI appears to take part in a broad range of physiological functions, and therefore drug safety might become a limiting factor for the development of drugs which interfere with enzymatic activity of extracellular PDI.

## Supporting Information

Figure S1 **Effect of NSDV infection on the cellular cytoskeleton**. Vero cells were infected with NSDVi at a MOI of 0.3. After 16 h, cells were fixed using 3% PFA and (a–f) stained using Alexa Fluor 488 Phalloidin (green; actin) and rabbit anti-N, followed by Alexa Fluor 568 goat anti-rabbit IgG (red); (g–o) cells were opened with ice-cold methanol and stained using mouse anti-α-tubulin antibody and rabbit anti-N, followed by Alexa Fluor 488 goat anti-mouse IgG (green) and Alexa Fluor 568 goat anti-rabbit IgG (red). Nuclei were counterstained using DAPI (blue). Dashed boxes in a–c and g–i indicate the source of the enlarged areas shown in d–f and j–l, respectively.
(TIF)

Figure S2 **Effect of NSDV infection on PDI in human and bovine cell lines.** A549 (human lung) cells (a–c) or BFA (bovine foetal aortic endothelial) cells (d–i) were infected with NSDVi at a MOI of 0.3. After 16 h (a–c) or 72 h (d–f) cells were fixed and stained using specific antibodies. (a–f) Cells were fixed using 3% PFA followed by ice-cold methanol, and then stained with mouse anti-PDI (clone 1D3) and rabbit anti-N. (g–i) Cells fixed with 3% PFA only, labelled with mouse anti-PDI (clone 1D3), then again fixed with 3% PFA, opened with ice-cold methanol and stained with rabbit anti-N. Proteins were visualised using Alexa Fluor 488 goat anti-mouse IgG (green) and Alexa Fluor 568 goat anti-rabbit IgG (red). Nuclei were counterstained using DAPI (blue). Bars correspond to 40 μm.
(TIF)

Figure S3 **Time course of changes to ERp57 in NSDV-infected cells.** Vero cells were infected with NSDVi at a MOI of 6 and fixed at 8, 12 and 16 hpi. (a–i) Cells were fixed using 3% PFA followed by ice cold methanol and stained with rabbit anti-ERp57 antibody and mouse anti-PreGn. (j–l) cells were fixed with 3% PFA, labelled with rabbit anti-ERp57 antibody, again fixed with 3% PFA, opened with ice-cold methanol, and labelled using mouse anti-PreGn. Proteins were visualised with Alexa Fluor 488 goat anti-mouse IgG (green) and Alexa Fluor 568 goat anti-rabbit IgG (red). Nuclei were counterstained using DAPI (blue). Bars correspond to 16 μm.
(TIF)

Figure S4 **Differences in the expression levels of N and PreGn in NSDV-infected cells**. Samples were prepared as for Figure 1 except that cells were stained with rabbit anti-N protein and mouse anti-PreGn antibody. Proteins were visualised using Alexa Fluor 488 goat anti-mouse IgG (green) and Alexa Fluor 568 goat anti-rabbit IgG (red). Nuclei were counterstained using DAPI (blue). Bars correspond to 40 μm. Arrows indicate cells where N but not PreGn was detected.
(TIF)

Figure S5 **Localisation of plasmid-expressed NSDV glycoproteins**. Vero were transfected with 1 μg of pCAGGs_MCSII_PreGn_V5 (a–c), pCAGGs_MCSII_PreGc_V5 (d–f) or pCAGGs_MCSII_NSM_V5 (g–i). After 24 h, cells were fixed with 3% PFA followed by ice cold methanol, and incubated with mouse anti-GM130 (cisGolgi) antibody, followed by Alexa Fluor 568 goat anti-mouse IgG (red). Then plasmid-expressed proteins were visualised with anti-V5 antibody conjugated to Alexa Fluor 488 (green). Nuclei were counterstained using DAPI (blue). Bars correspond to 20 μm.
(TIF)

Figure S6 **The effect of NSDV protein expression on PDI.** Vero cells were transfected with 1 μg of pcDNA6-GV-N (a–c) or pcDNA6-GV-La (d–f). After 24 h, cells were fixed with 3% PFA followed by ice-cold methanol, and were stained using mouse anti-PDI (clone 1D3) and rabbit anti-N (a–c) or rabbit anti-L (d–f). Proteins were visualised with Alexa Fluor 488 goat anti-mouse IgG (green) and Alexa Fluor 568 goat anti-rabbit IgG (red). Nuclei were counterstained using DAPI (blue). Bars correspond to 40 μm.
(TIF)
