# Supplementary figures and images for: The Nairovirus Nairobi Sheep Disease Virus/Ganjam Virus Induces the Translocation of Protein Disulphide Isomerase-Like Oxidoreductases from the Endoplasmic Reticulum to the Cell Surface and the Extracellular Space
Source: PLoS One. 2014 Apr 8;9(4):e94656. doi: 10.1371/journal.pone.0094656 (PMC3979861; doi:10.1371/journal.pone.0094656)

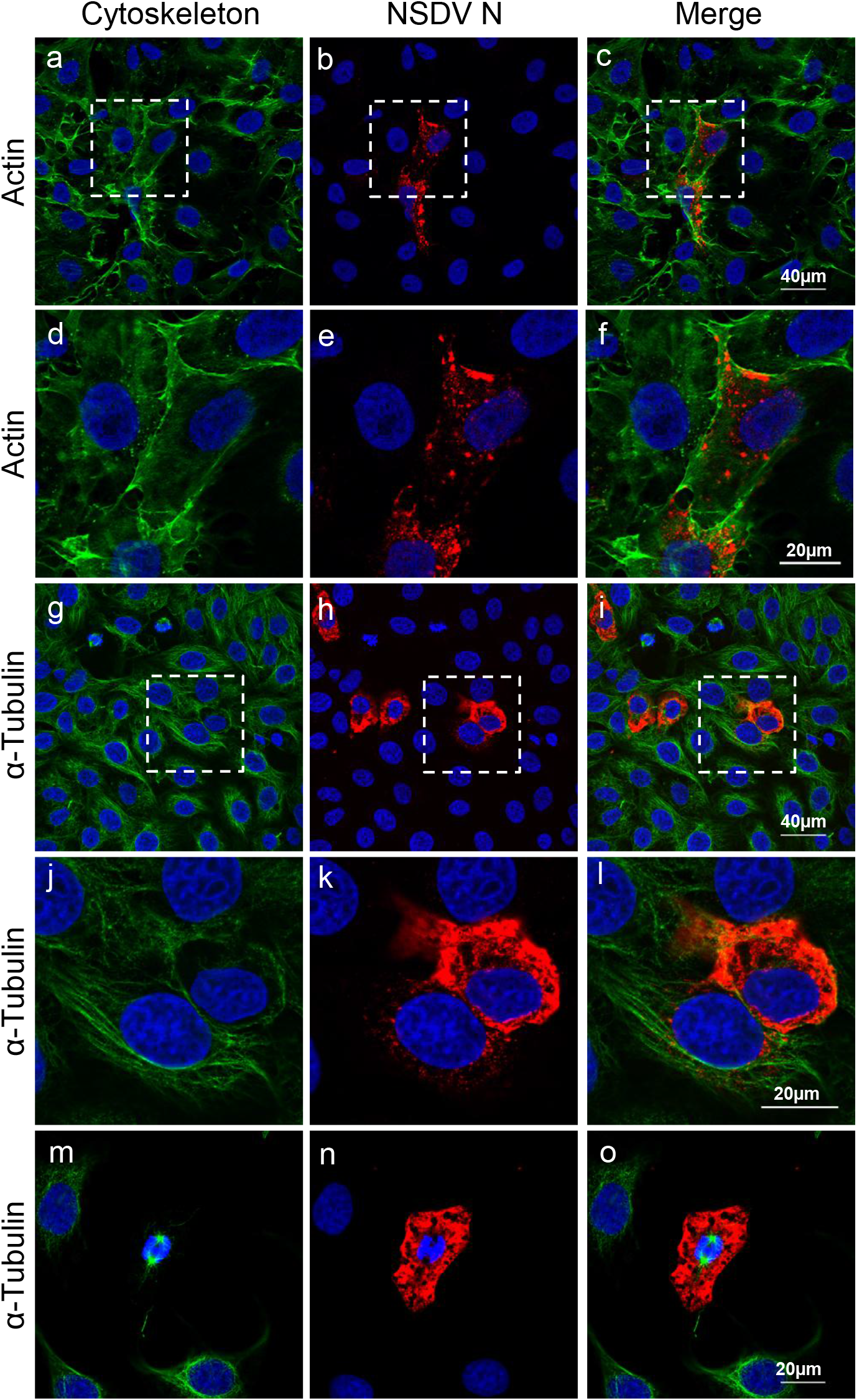

Supplement: Figure S1 — Effect of NSDV infection on the cellular cytoskeleton. Vero cells were infected with NSDVi at a MOI of 0.3. After 16 h, cells were fixed using 3% PFA and (a–f) stained using Alexa Fluor 488 Phalloidin (green; actin) and rabbit anti-N, followed by Alexa Fluor 568 goat anti-rabbit IgG (red); (g–o) cells were opened with ice-cold methanol and stained using mouse anti-α-tubulin antibody and rabbit anti-N, followed by Alexa Fluor 488 goat anti-mouse IgG (green) and Alexa Fluor 568 goat anti-rabbit IgG (red). Nuclei were counterstained using DAPI (blue). Dashed boxes in a–c and g–i indicate the source of the enlarged areas shown in d–f and j–l, respectively. (TIF) [file pone.0094656.s001.tif]

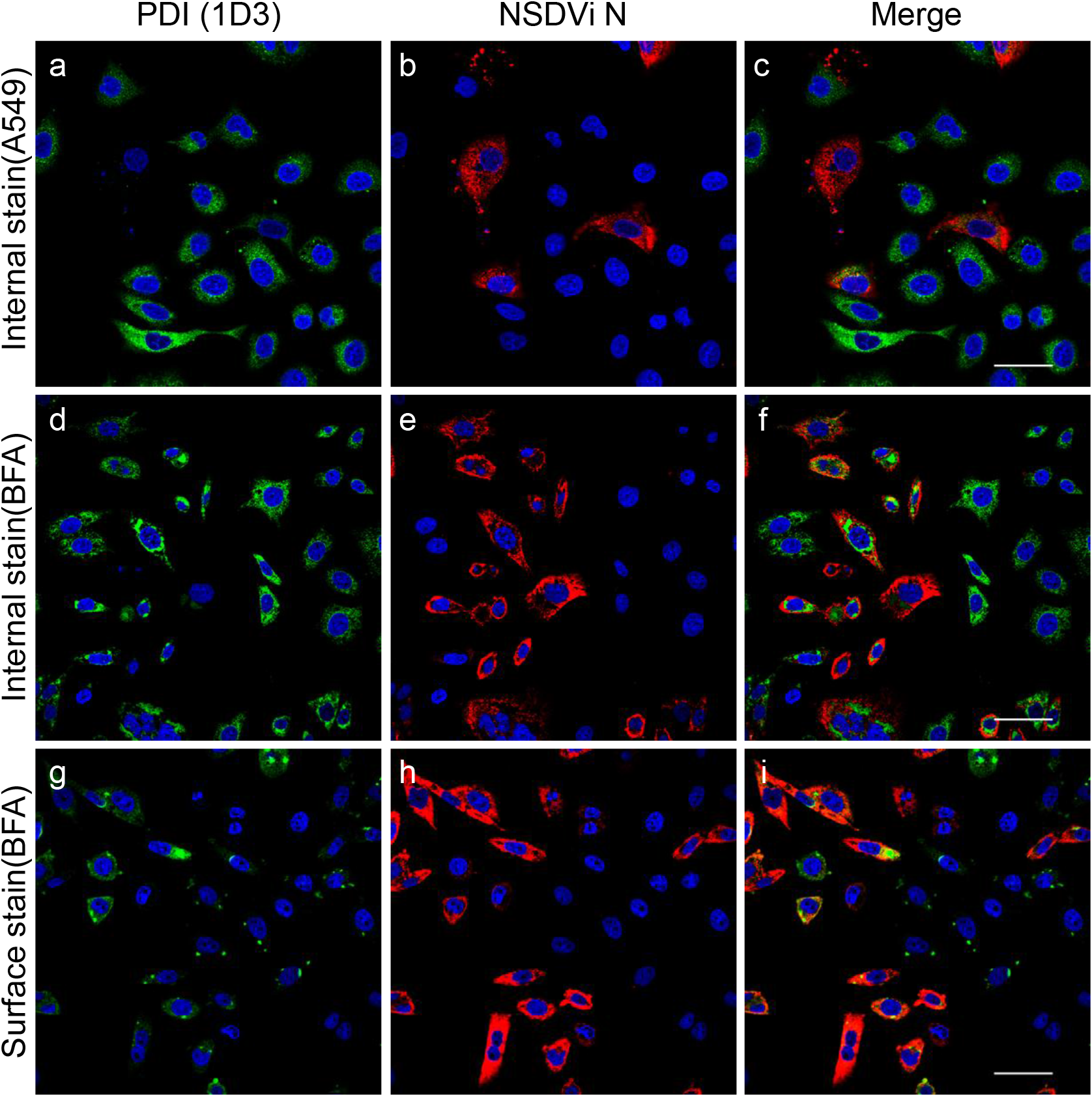

Supplement: Figure S2 — Effect of NSDV infection on PDI in human and bovine cell lines. A549 (human lung) cells (a–c) or BFA (bovine foetal aortic endothelial) cells (d–i) were infected with NSDVi at a MOI of 0.3. After 16 h (a–c) or 72 h (d–f) cells were fixed and stained using specific antibodies. (a–f) Cells were fixed using 3% PFA followed by ice-cold methanol, and then stained with mouse anti-PDI (clone 1D3) and rabbit anti-N. (g–i) Cells fixed with 3% PFA only, labelled with mouse anti-PDI (clone 1D3), then again fixed with 3% PFA, opened with ice-cold methanol and stained with rabbit anti-N. Proteins were visualised using Alexa Fluor 488 goat anti-mouse IgG (green) and Alexa Fluor 568 goat anti-rabbit IgG (red). Nuclei were counterstained using DAPI (blue). Bars correspond to 40 μm. (TIF) [file pone.0094656.s002.tif]

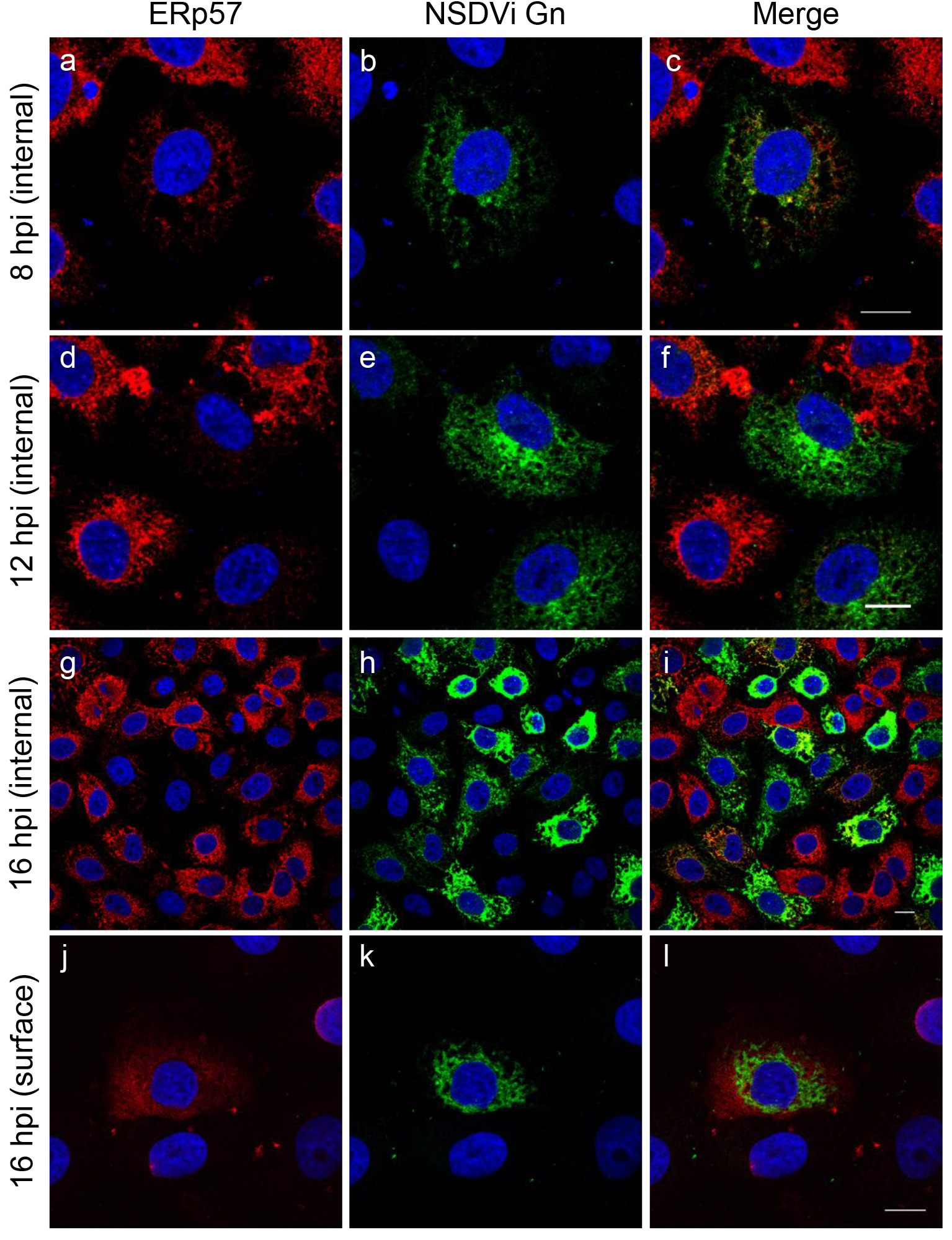

Supplement: Figure S3 — Time course of changes to ERp57 in NSDV-infected cells. Vero cells were infected with NSDVi at a MOI of 6 and fixed at 8, 12 and 16 hpi. (a–i) Cells were fixed using 3% PFA followed by ice cold methanol and stained with rabbit anti-ERp57 antibody and mouse anti-PreGn. (j–l) cells were fixed with 3% PFA, labelled with rabbit anti-ERp57 antibody, again fixed with 3% PFA, opened with ice-cold methanol, and labelled using mouse anti-PreGn. Proteins were visualised with Alexa Fluor 488 goat anti-mouse IgG (green) and Alexa Fluor 568 goat anti-rabbit IgG (red). Nuclei were counterstained using DAPI (blue). Bars correspond to 16 μm. (TIF) [file pone.0094656.s003.tif]

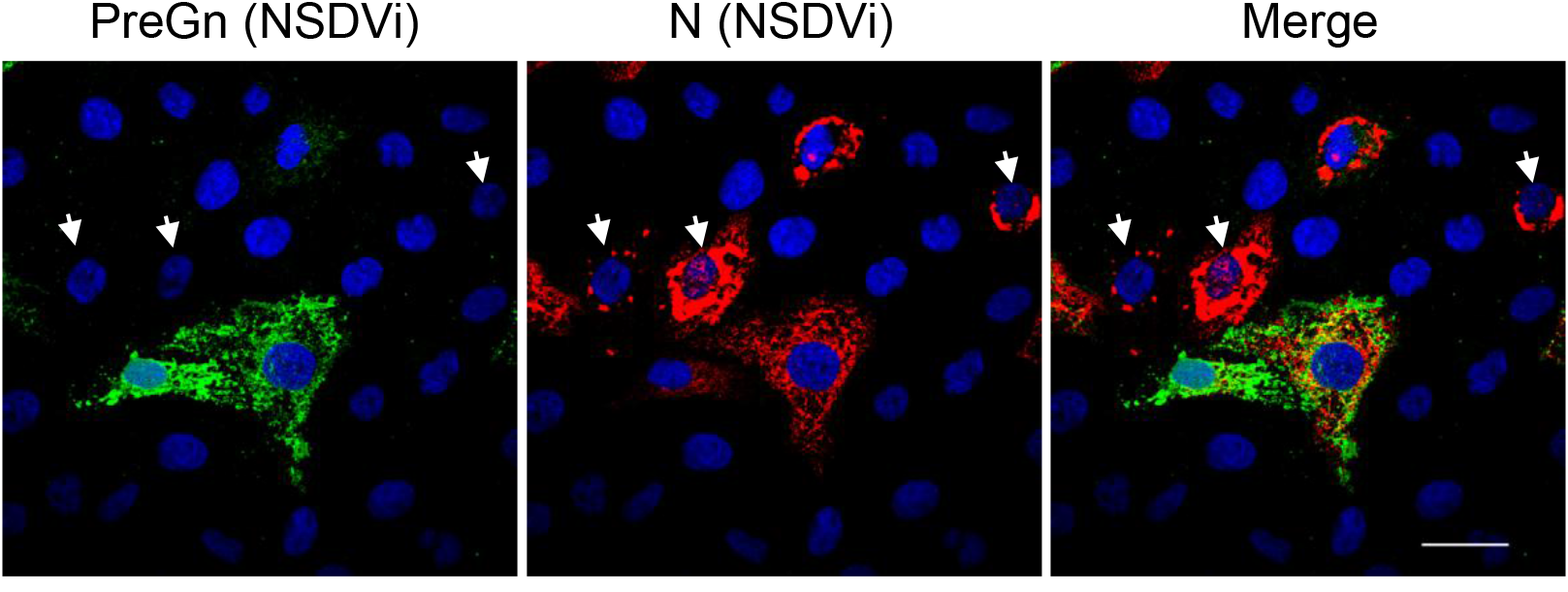

Supplement: Figure S4 — Differences in the expression levels of N and PreGn in NSDV-infected cells. Samples were prepared as for Figure 1 except that cells were stained with rabbit anti-N protein and mouse anti-PreGn antibody. Proteins were visualised using Alexa Fluor 488 goat anti-mouse IgG (green) and Alexa Fluor 568 goat anti-rabbit IgG (red). Nuclei were counterstained using DAPI (blue). Bars correspond to 40 μm. Arrows indicate cells where N but not PreGn was detected. (TIF) [file pone.0094656.s004.tif]

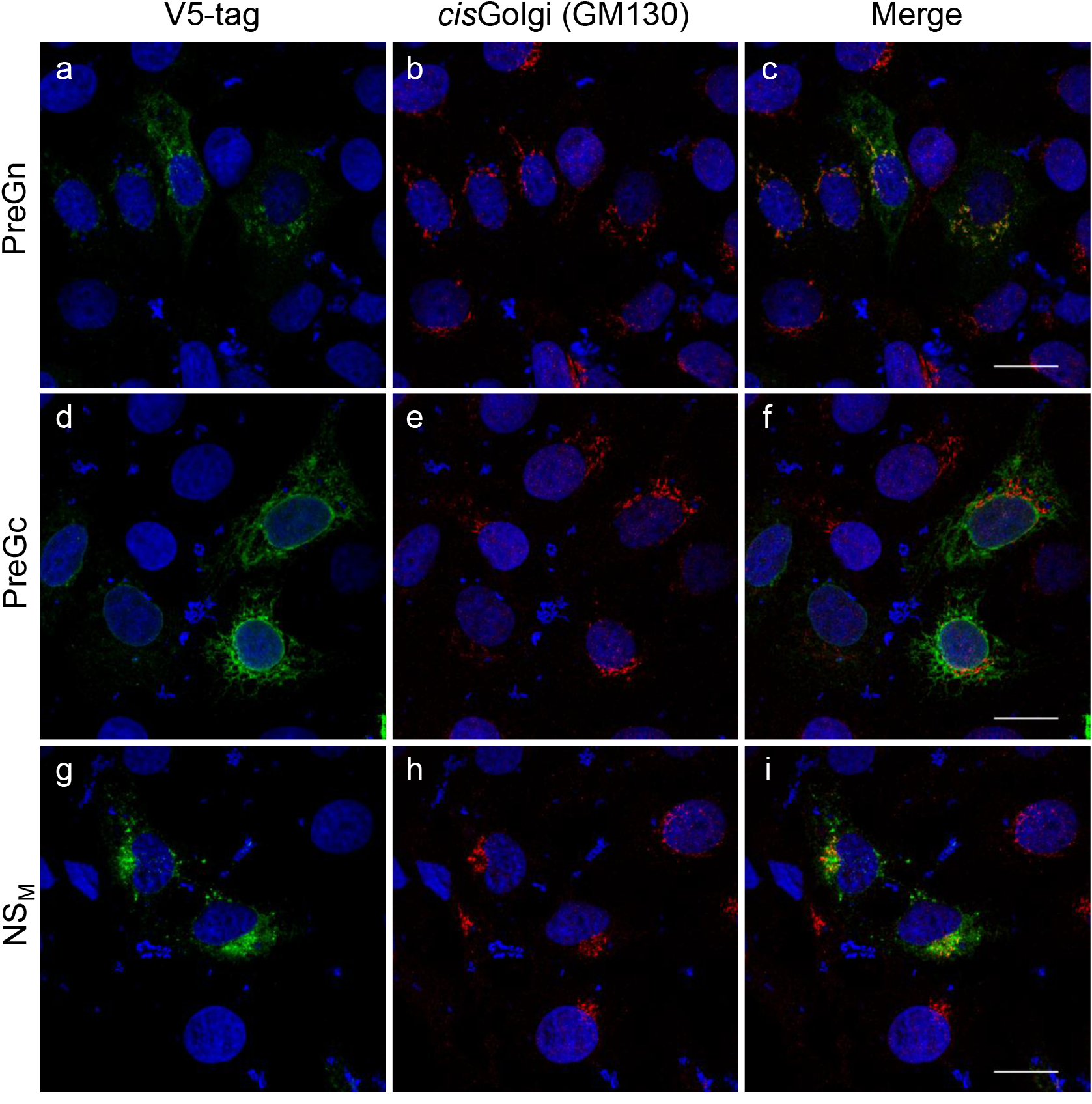

Supplement: Figure S5 — Localisation of plasmid-expressed NSDV glycoproteins. Vero were transfected with 1 μg of pCAGGs_MCSII_PreGn_V5 (a–c), pCAGGs_MCSII_PreGc_V5 (d–f) or pCAGGs_MCSII_NSM_V5 (g–i). After 24 h, cells were fixed with 3% PFA followed by ice cold methanol, and incubated with mouse anti-GM130 (cisGolgi) antibody, followed by Alexa Fluor 568 goat anti-mouse IgG (red). Then plasmid-expressed proteins were visualised with anti-V5 antibody conjugated to Alexa Fluor 488 (green). Nuclei were counterstained using DAPI (blue). Bars correspond to 20 μm. (TIF) [file pone.0094656.s005.tif]

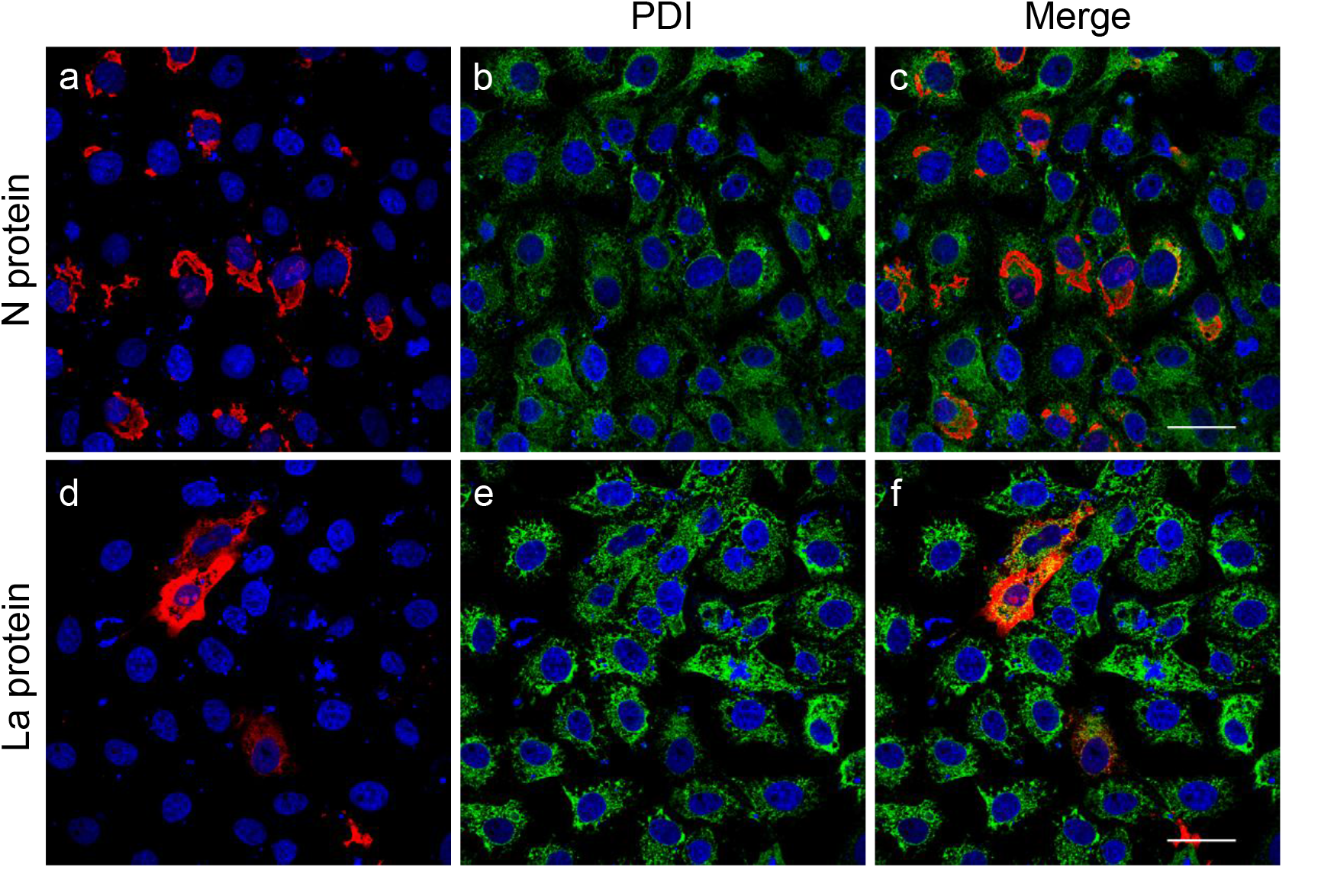

Supplement: Figure S6 — The effect of NSDV protein expression on PDI. Vero cells were transfected with 1 μg of pcDNA6-GV-N (a–c) or pcDNA6-GV-La (d–f). After 24 h, cells were fixed with 3% PFA followed by ice-cold methanol, and were stained using mouse anti-PDI (clone 1D3) and rabbit anti-N (a–c) or rabbit anti-L (d–f). Proteins were visualised with Alexa Fluor 488 goat anti-mouse IgG (green) and Alexa Fluor 568 goat anti-rabbit IgG (red). Nuclei were counterstained using DAPI (blue). Bars correspond to 40 μm. (TIF) [file pone.0094656.s006.tif]
